# Agent-specific learning signals for self–other distinction during mentalising

**DOI:** 10.1371/journal.pbio.2004752

**Published:** 2018-04-24

**Authors:** Sam Ereira, Raymond J. Dolan, Zeb Kurth-Nelson

**Affiliations:** 1 Max Planck UCL Centre for Computational Psychiatry and Ageing Research, UCL, London, United Kingdom; 2 Wellcome Centre for Human Neuroimaging, UCL, London, United Kingdom; 3 Google DeepMind, London, United Kingdom; University of Cambridge, United Kingdom of Great Britain and Northern Ireland

## Abstract

Humans have a remarkable ability to simulate the minds of others. How the brain distinguishes between mental states attributed to self and mental states attributed to someone else is unknown. Here, we investigated how fundamental neural learning signals are selectively attributed to different agents. Specifically, we asked whether learning signals are encoded in agent-specific neural patterns or whether a self–other distinction depends on encoding agent identity separately from this learning signal. To examine this, we tasked subjects to learn continuously 2 models of the same environment, such that one was selectively attributed to self and the other was selectively attributed to another agent. Combining computational modelling with magnetoencephalography (MEG) enabled us to track neural representations of prediction errors (PEs) and beliefs attributed to self, and of simulated PEs and beliefs attributed to another agent. We found that the representational pattern of a PE reliably predicts the identity of the agent to whom the signal is attributed, consistent with a neural self–other distinction implemented via agent-specific learning signals. Strikingly, subjects exhibiting a weaker neural self–other distinction also had a reduced behavioural capacity for self–other distinction and displayed more marked subclinical psychopathological traits. The neural self–other distinction was also modulated by social context, evidenced in a significantly reduced decoding of agent identity in a nonsocial control task. Thus, we show that self–other distinction is realised through an encoding of agent identity intrinsic to fundamental learning signals. The observation that the fidelity of this encoding predicts psychopathological traits is of interest as a potential neurocomputational psychiatric biomarker.

## Author summary

In order for people to have meaningful social interactions, they need to infer each other’s beliefs. Converging evidence from humans and nonhuman primates suggests that a person’s brain can represent a second person’s beliefs by simulating that second person’s brain activity. However, it is not known how the outputs of those simulations are identified as ‘yours and not mine’. This ability to distinguish self from other is required for social cognition, and it may be impaired in mental health disorders with social cognitive deficits. We investigated self–other distinction in healthy adults learning about an environment both from their own point of view and the point of view of another person. We used computationally identified learning variables and then detected how these variables are represented by measuring magnetic fields in the brain. We found that the human brain can distinguish self from other by expressing these signals in dissociable activity patterns. Subjects who showed the largest difference between self signals and other signals were better at distinguishing self from other in the task and also showed fewer traits of mental health disorders.

## Introduction

Social interactions are underpinned by an ability to infer the mental states of self and others, referred to as mentalising [1]. The discovery of mirror neurons in the macaque premotor cortex [2] introduced the notion that in mentalising, the primate brain might directly simulate another agent’s cognitive process. More recently, functional magnetic resonance imaging (fMRI) studies [3–8] and intracranial recordings [9] in humans, as well as single-cell recordings in monkeys [10], have shown that when a subject observes another agent interact with its environment, the subject’s brain encodes not only the other agent’s motor activity but also their reward prediction errors (RPEs). In other words, subjects appear to simulate the reinforcement learning processes of other agents.

These simulated learning signals localise to specific cortical regions, such as the anterior cingulate gyrus [9–11]. A functional segregation of learning signals can allow the brain to encode information about whether learning is arising out of the individual’s own behavioural interactions with the environment or whether learning is taking place vicariously through observing the behaviour of another agent. In a similar vein, it has been suggested that the medial prefrontal cortex (mPFC) supports a functional axis that encodes whether behaviour is executed or imagined [12, 13].

For simulation to be useful in social interactions, the brain must discriminate signals attributed to self from simulated signals attributed to other agents [14–17]. An impairment in this self–other distinction is a defining feature of autism spectrum disorder [18–21]. Similar impairments have also been reported in conditions such as schizophrenia [22, 23], psychopathy [17], and borderline personality disorder [24]. An aberrant self–other distinction might also underpin the social dysfunction seen in psychopathologies, including depression [25, 26] and addiction [27–29].

A prefrontal coding scheme that discriminates between instrumental and observational learning, or executed and imagined behaviour, could provide a useful heuristic for a self–other distinction but would be insufficient for discriminating amongst signals attributed to different agents as a general-purpose computation. For instance, the false belief task [30], a standard test of mentalising ability, requires that subjects make inferences about an environment and then selectively attribute one belief-state to self and a different belief-state to another agent for whom the environment is only partially observable. These belief-states are not informed by the behaviour of the subject or the other agent but arise through passively observing the environment. In this case, neural coding schemes that discriminate between executed behaviour and observed or imagined behaviour cannot facilitate a self–other distinction, and a more fundamental computation for selectively attributing signals to different agents is required.

Thus, an open question for simulation theory is how self–other distinction is achieved [16]. If inferring another agent’s mental state requires the brain to simulate that agent’s computations, how are the outputs of those computations identified as ‘belonging to other’? One possibility is that variables for learning and decision-making are encoded in distinct neural activity patterns, depending on the agent to whom these signals are attributed. Such architecture would entail an encoding of agent identity intrinsic to representations of these low-level signals. A second possibility is that a learning signal is always encoded in an agent-independent pattern. In this case, the learning signal and the identity of the agent to whom the signal is attributed would need to be encoded in 2 separate activity patterns.

Here, we test whether learning signals are encoded in agent-specific activity patterns, and thus whether self–other distinction requires agent identity to be encoded separately from a low-level learning signal. We used a novel paradigm inspired by false belief tasks, in which subjects learned about a fluctuating state in the environment. In so doing, they were also required to intermittently switch their frame of reference between self and other. The 2 agents (self and other) received different information such that their belief trajectories were uncorrelated, enabling us to measure self-attributed and other-attributed learning signals independently. Unlike previous paradigms eliciting simulated signals, subjects did not observe the agent’s behaviour, and there was no reinforcement of learning by either reward or punishment. Learning for self and learning for other thus recruited the same input channels and were identically salient and identically motivating. The task design rules out a potential confound of simulated reward learning [3–8] wherein self-attributed reward-related decision signals (such as RPEs) pertain to rewards expected to be received by the participant, while other-attributed reward-related decision signals do not. We measured the neural encoding of learning signals using magnetoencephalography (MEG) in order to measure whether the representations of these signals are agent-specific and also how agent-specificity evolves over the time course of a single trial. Of note, our task design required sparse probe trials and therefore a larger total number of trials than would be possible to acquire in a single fMRI session.

## Results

### Behaviour

We present data from 38 healthy adults (see Materials and methods for participant details). During MEG scanning, they observed a sequence of samples from a Bernoulli distribution, with a drifting Bernoulli parameter P. This is the probability, on each trial, of seeing 1 of 2 possible outcomes. Another participant, who sat outside the scanner in a different room, played the exact same game (see Materials and methods). This other subject was able to observe some of the samples seen by the scanned participant (‘shared’ trials) but not all of them (‘privileged’ trials). Additionally, the nonscanned participant was occasionally presented with misleading samples (‘decoy’ trials). Therefore, the nonscanned participant sampled evidence that induced a false belief about P (P_fb_). These 3 types of trial (‘privileged’, ‘shared’, and ‘decoy’) were balanced in frequency and distributed evenly throughout the task in a pseudorandom order.

**Fig 1:**
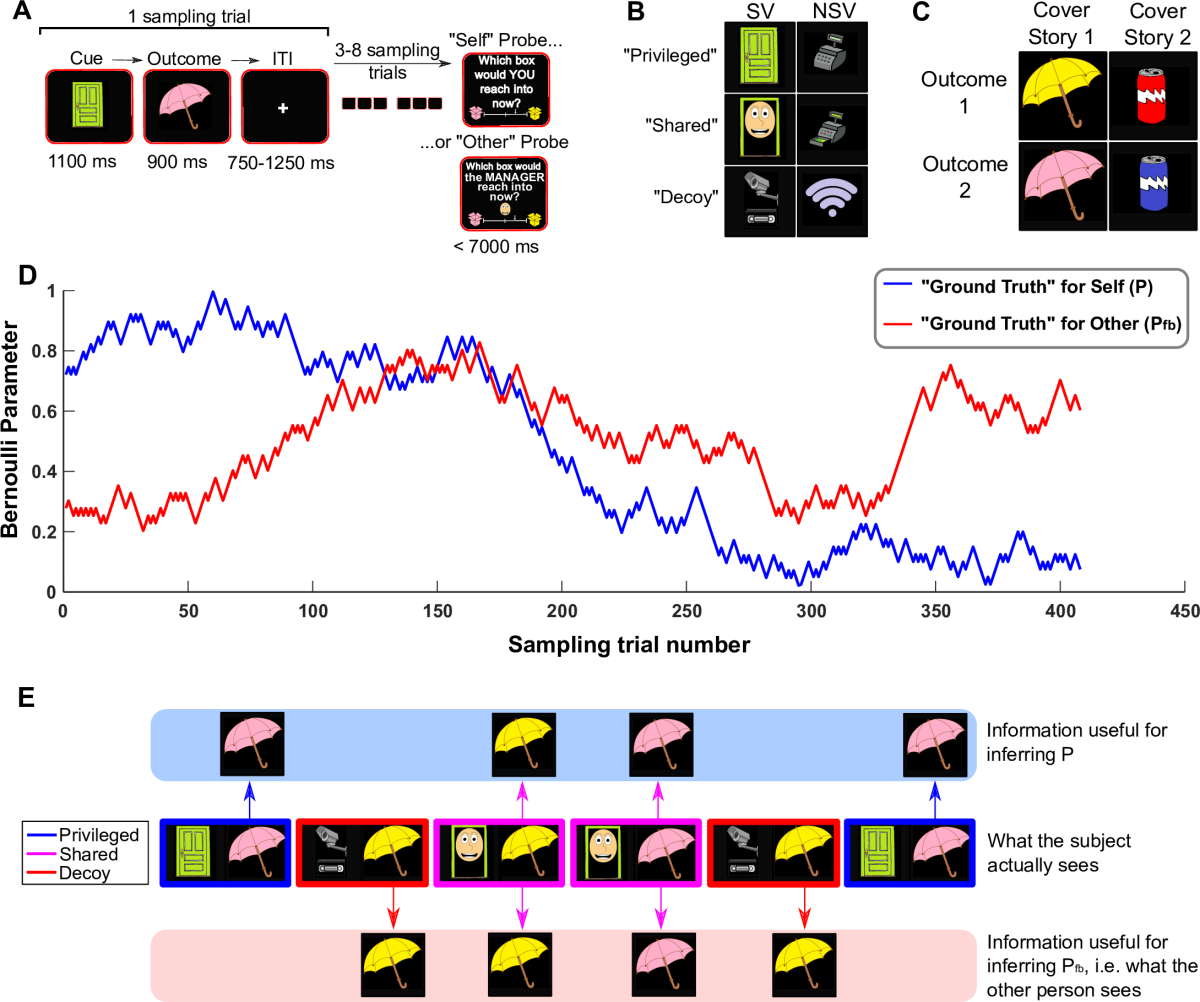
Behavioural paradigm. (A) An example of a sampling trial and probe trial. Sampling trials consisted of a cue (‘privileged’, ‘shared’, or ‘decoy’) followed by a Bernoulli outcome (heads or tails), followed by a variable ITI. After 4 to 9 sampling trials, subjects were probed with either a ‘self’ or an ‘other’ probe trial, in which they had to report their own belief or their estimate of the other agent’s belief about the current probability of seeing one outcome over the other, in this case a yellow sun-shade signifying sunshine and pink umbrella signifying rain, by moving an arrow along a continuous scale ranging from 0 (certain for one outcome) to 1 (certain for the other outcome). (B) The set of images used as cues for the SV and NSV. (C) The set of images used as outcomes for cover story 1 and cover story 2. (D) An example pair of uncorrelated random walks used to generate a full trial sequence. Samples were drawn from these generative Bernoulli distributions to produce 2 sequences of ‘coin flips’, one for self (blue) and one for other (red). (E) An exemplar sequence of 6 sampling trials in the SV with cover story 1. This schematic demonstrates how different information can be used to infer P and P_fb_. In ‘privileged’ trials (blue squares and arrows), only the subject sees the information. In ‘shared’ trials (magenta squares and arrows), both agents receive the information. In ‘decoy’ trials (red squares and arrows), both agents receive information, but the scanned subject knows that it is misleading whilst the other agent does not. The 3 trial types were balanced in frequency and occurred in pseudorandom order. ITI, intertrial interval; NSV, nonsocial version; P, Bernoulli parameter; P_fb_, the other agent’s false belief about the Bernoulli parameter; SV, social version.

In a version of the game we refer to as the social version (SV), ‘privileged’ and ‘decoy’ trials were signalled to the scanned participant, who thus had access to information about both P and P_fb_. On ‘self’ probe trials, the scanned participant was required to report their estimate of P, by positioning an arrow along a virtual continuous scale that ranged from a probability of 0 (certain to see one outcome) to 1 (certain to see the other outcome). On ‘other’ probe trials, the scanned participant had to put themselves in the shoes of the nonscanned participant and report their estimate of P_fb_. Crucially, the information that the scanned subject used to compute P_fb_ was sampled at the same rate as the information used to compute P. We refer to the subject’s belief about P as B, and we refer to their belief about P_fb_ as B_fb_. The structure of sampling trials and probe trials is outlined in Fig 1A.

All subjects also played a nonsocial version (NSV) of this game, which did not involve another participant. Here, the scanned participant had to keep track of the belief-state of a fictional ‘computer’ that received limited and misleading information and stored a false estimate of P (P_fb_). On ‘other’ probe trials in the NSV, the scanned participant was asked to imagine themselves in a counterfactual situation, wherein they acted using the false information provided by the computer. Thus, in the SV, participants switched their frame of reference between self and other, whilst in the NSV, participants switched their frame of reference between self and a counterfactual self. The only structural differences between the SV and NSV pertained to the cover stories, the images used for stimuli, and the wording of the ‘other’ probe trials (see Materials and methods). Note that for both the SV and NSV, we pregenerated trial sequences that minimised correlation between the belief trajectories of the 2 agents (Fig 1D, Fig 1E; also see Materials and methods).

For each subject, we assessed behavioural accuracy independently for the 2 versions of the task (SV and NSV) as well as on the 2 types of probe trial (‘self’ and ‘other’). This resulted in 4 conditions overall, for which accuracy was defined relative to chance performance (see Materials and methods). Where an accuracy of 0 is equivalent to chance-level performance, mean accuracies (with SDs) for the SV were 0.11 (0.04) in ‘self’ probe trials and 0.11 (0.04) in ‘other’ probe trials. For the NSV, these were 0.11 (0.04) for ‘self’ probe trials and 0.10 (0.05) for ‘other’ probe trials (see S1 Fig). The group performed significantly better than chance in all 4 conditions as assessed with 4 separate one-sample *t* tests on the mean accuracies per subject (*P* < 0.0001 in all 4 conditions). There were no differences in accuracy between the 2 probe trial types, or between the 2 versions of the game (ANOVA: main effect of probe trial type: F[1, 148] = 1.54, *P* = 0.22; main effect of game version: F[1, 148] = 0, *P* = 0.96; interaction: F[1, 148] = 0.06, *P* = 0.81). Thus, all 4 conditions were similar in difficulty.

### Modelling simulated belief updates with simulated PEs

We fitted 21 models to the probe trial behaviour of each subject, separately for the SV and NSV. There were 3 principal groups of model (see Table 1 for a summary and Materials and methods for details). Group A models assumed that subjects’ beliefs were constructed from an average over recently sampled information. Group B models were based on an assumption of Rescorla-Wagner (RW) updating [31], in which the models derive prediction errors (PEs) on each trial from the difference between the actual and expected outcomes. PE^s^ updated the beliefs of self, while PE^o^ was a simulation of the other agent’s PE, for updating the beliefs of other in the SV or ‘counterfactual self’ in the NSV. A subset of group B models also included ‘leak’ parameters that allowed PE signals to erroneously update the wrong agent’s belief, thus capturing an inability to maintain separate belief updates for the 2 agents. All group B models also assumed that the PE^s^ had a value of 0 on ‘decoy’ trials whilst PE^o^ had a value of 0 on ‘privileged’ trials. Group C models were like group B models except that they did not make this assumption; instead, they allowed PE^s^ and PE^o^ to update the beliefs of self and other, respectively, in all 3 trial types.

**Table 1:** Summary of all models fitted to behavioural data.

Model number	Model group	α	τ	δ	λ	Total number of free parameters
1	A		1			1
2	A		1			1
3	B	1	1			2
4	B	1	1	1		3
5	B	2	1	1		4
6	B	2	2	1		5
7	B	2	2	2		6
8	B	1	2	1		4
9	B	1	1	2		4
10	B	2	1	2		5
11	B	2	2	2	2	8
12	B	2	2	2	1 (shared)	7
13	B	2	2	2	1 (PE^o^ updates B)	7
14	B	2	2	2	1 (PE^s^ updates B_fb_)	7
15	B	2	1	1	2	6
16	B	1	1	1	1 (shared)	4
17	B	1	1	1	1 (PE^o^ updates B)	4
18	B	1	1	1	1 (PE^s^ updates B_fb_)	4
19	B	1	1	1	2	5
20	C	1	1	1		3
21	C	1	1	1		3

Twenty-one models were evaluated in total. The table illustrates the differences between these models by identifying the groups A–C and displaying which parameters were included in each model. In the α, τ, and δ columns, a ‘1’ indicates a parameter shared between the update equations for the 2 agents. A ‘2’ indicates that 2 different values of that parameter were fitted for each of the 2 update equations. In the λ column, a ‘2’ indicates that 2 different leak parameters were fitted, one for each update equation, allowing for an asymmetrical bidirectional leak. A ‘1 (shared)’ indicates that 1 leak parameter was shared between both equations to allow for a symmetrical bidirectional leak. Alternatively, 1 parameter was included in only 1 equation to allow for a unidirectional leak. The direction of any unidirectional leak is described in the table. If the table cell is empty, it indicates that the relevant parameter was not included in that model.

Abbreviations: B, belief about the Bernoulli parameter; B_fb_, belief about the other agent’s false belief about the Bernoulli parameter; PE^o^, other-attributed prediction error; PE^s^, self-attributed prediction error.

**Fig 2:**
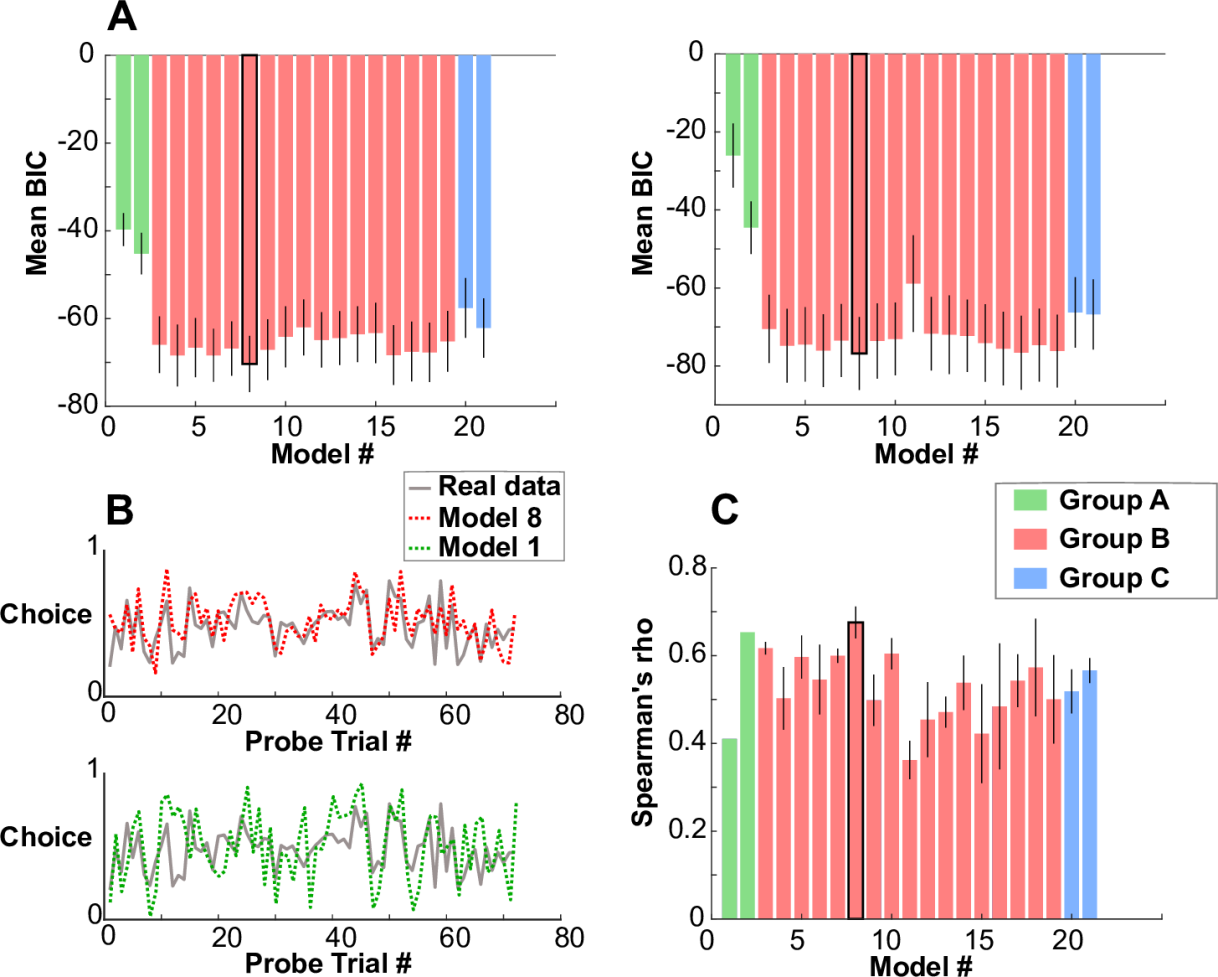
Behavioural analysis using computational models. (A) Results from a Bayesian model comparison for the SV (left) and NSV (right). The bar representing the winning model in both versions of the game is highlighted in bold (model 8). Error bars show SEM. The winning model in both cases is a 4-parameter model that incorporates 2 types of PE signal, each one attributed to a different agent. (B) Generative performance of the winning model (model 8) compared to the generative performance of a less successful model (model 1). In the top panel, we used model 8 to simulate choice data for 1 subject. In the bottom panel, we used model 1 to simulate choice data for the same subject. To select which subject to use for this display, we computed the median BIC score of the model 8 fits to the SV data and selected the subject whose fit was closest to this value. Thus, the subject can be considered an ‘average’ subject in terms of goodness-of-fit of the winning model. The simulated data (red) is qualitatively similar to the subject’s real choice data. (C) Consistency of each model between the 2 games. Each bar shows, for a different model, how correlated the subjects’ parameter estimates fitted to the SV data are to subjects’ parameter estimates fitted to the NSV data. The model with the highest Spearman coefficient (averaged across parameters) is highlighted in bold. This was again model 8, the very same model with the best average predictive performance for the SV and NSV independently. Note that model 1 and model 2 do not have error bars because these models both contained a single parameter, and so only 1 correlation coefficient was computed for both of these models. Error bars show SEM. See S2, S3 and S4 Figs for further model comparisons and parameter recovery. See S1 Data for all numerical values. BIC, Bayesian Information Criterion; NSV, nonsocial version; PE, prediction error; SV, social version.

We compared models separately for the SV and NSV using the Bayesian Information Criterion (BIC). For both the SV and NSV, model 8 had the lowest mean BIC value (Fig 2A). This model incorporated 2 separate PE signals and included 4 free parameters: a learning rate (α) regulated the update of the beliefs of the 2 agents, a memory decay parameter (δ) controlled the rate of ‘forgetting’ for the beliefs of the 2 agents, and 2 temperature parameters (τ_s_, τ_o_) governed choice stochasticity on ‘self’ probe trials and ‘other’ probe trials, respectively (see S3 Fig for parameter recovery). This model generated synthetic choice data qualitatively similar to subjects’ real choice data (Fig 2B). Noting large intersubject variability in BIC values, we also employed a random-effects Bayesian model selection [32] to compare the winning model with the second best model (S4 Fig) and found, for both the SV and NSV, an exceedance probability in excess of 0.99. This is the probability that the winning model better explains a randomly chosen subject’s data.

We also assessed the correlation between parameter estimates fitted to the SV and parameter estimates fitted to the NSV (Fig 2C). For each model, we obtained a correlation coefficient for each parameter and then took the mean of those coefficients as a summary statistic for the between-game consistency of the model. Because parameter values were not normally distributed, we computed the nonparametric Spearman’s rank correlation coefficient. We found that model 8 also had the highest between-game consistency. Thus, this model captured consistent dispositions in subjects’ choice behaviour across the 2 games.

After fitting models to the behavioural data, we then had parameter estimates for each model and each subject. We used model 8 along with each subject’s parameters for this model to generate trial-wise estimates of latent PEs and beliefs, which we then used in subsequent analyses on the MEG data. Note that PEs and belief values generated by other models were very similar, and consequently, our findings were not sensitive to the selection of a particular model.

### Neural representations of PEs and simulated PEs

**Fig 3:**
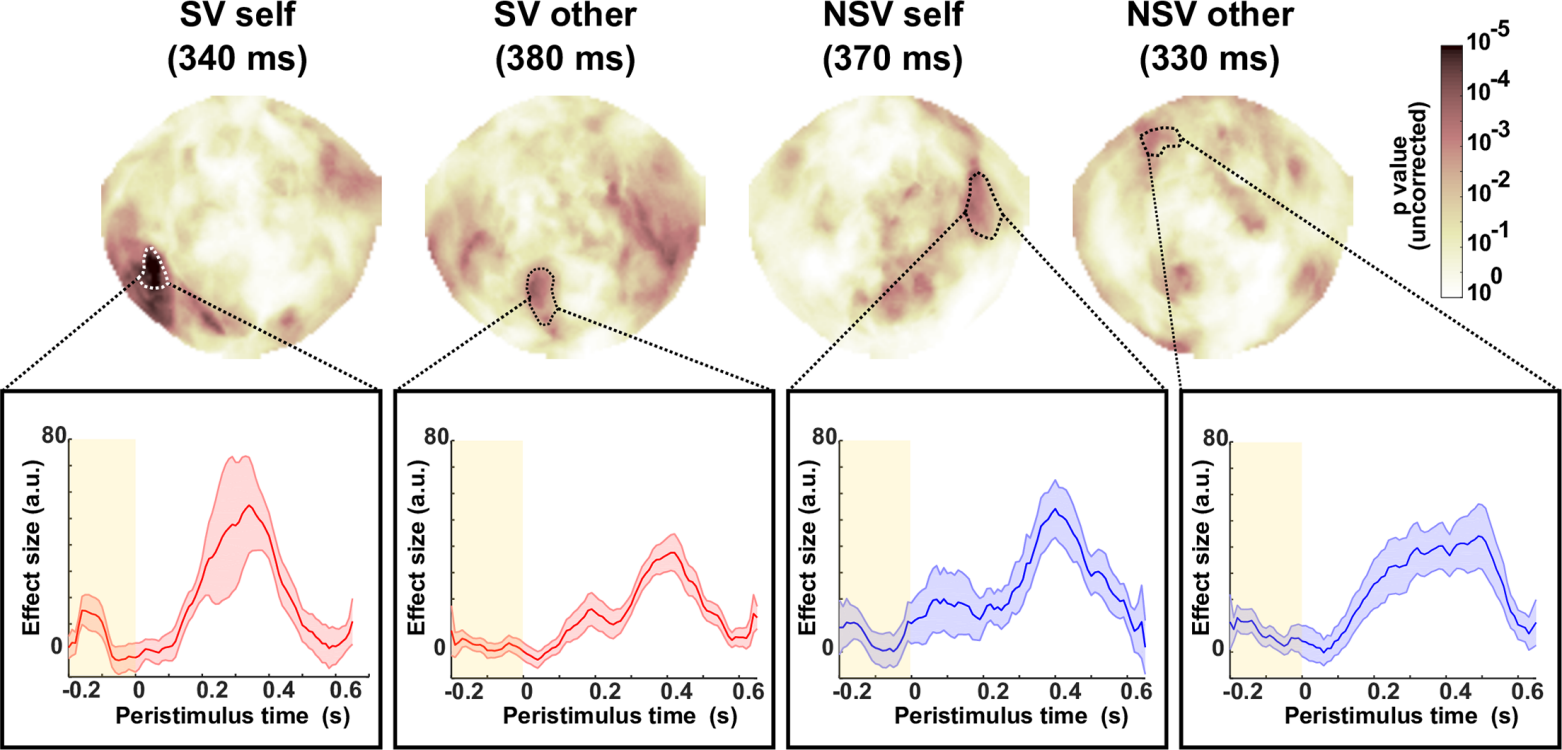
Regressing PE magnitude against MEG ERF. Group-level statistical maps projected onto scalp surface (frontal sensors towards top of page) after regressing |PE^s^| and |PE^o^| against the ERF for the SV and NSV. All 4 regressions yielded significantly large clusters that extended through multiple sensors and time points. The maps highlight the spatial extent of these clusters (dotted lines) at specific points in peristimulus time. The plots underneath show the temporal waveform (smoothed with a moving average filter with span 10) of the regression effect size from averaging over all spatial regions within the cluster. This has been averaged across subjects. The shaded regions show SEM. See S5 Fig for exemplar individual subjects. See S1 Data for all numerical values. ERF, event-related field; MEG, magnetoencephalography; NSV, nonsocial version; PE, prediction error; PE^s^, self-attributed prediction error; PE^o^, other-attributed prediction error; SV, social version.

We next asked whether |PE^s^| and |PE^o^| were encoded in the MEG signal, recorded during task performance, using a mass-univariate analysis (Fig 3). For each subject, we fit 2 separate linear regression models at each sensor and each peristimulus time point. We obtained trial-wise estimates of |PE^s^| and |PE^o^| using the winning model’s estimated free parameters fitted to the choice data. The first model regressed |PE^s^| against the event-related field (ERF) on ‘privileged’ and ‘shared’ sampling trials (i.e., trials in which PE^s^ was nonzero). The second regression model regressed |PE^o^| against ERFs on ‘decoy’ and ‘shared’ trials (i.e., trials in which PE^o^ was nonzero). This resulted in 4 statistical maps over sensors and time, 2 for the SV and 2 for the NSV.

We converted each of these maps into a 3D image (2 spatial dimensions and 1 temporal dimension) of baseline-corrected effect sizes (see Materials and methods). To make group-level inferences, we conducted a one-sided Wilcoxon signed-rank test at each pixel to determine whether the group median was significantly greater than 0. We thresholded the resulting 3D image with a cluster-forming threshold (*P* < 0.001) and identified clusters of contiguous suprathreshold pixels, which could extend through space and time.

We determined whether any clusters were significantly larger than chance with a nonparametric permutation test to generate null distributions of cluster extent. In each of the 4 regression models, we found clusters significantly larger than chance at a 0.05 family-wise error (FWE) level. In the SV, the clusters extended through parietal and occipital sensors, whilst in the NSV, the clusters extended through frontal and parietal sensors. For the SV PE^s^, the largest cluster extended from 330 ms to 390 ms and comprised 2,628 pixels (threshold 612). For the SV PE^o^, the largest cluster extended from 340 ms to 420 ms and comprised 2,032 pixels (threshold 624). For the NSV PE^s^, the largest cluster extended from 370 ms to 440 ms and comprised 1,621 pixels (threshold 554). For the NSV PE^o^, the largest cluster extended from 310 ms to 370 ms and comprised 847 pixels (threshold 569). Despite finding significant clusters at the group level, we also noted large intersubject differences in these spatiotemporal patterns (e.g., S5 Fig).

### Decoding agent identity from learning signals

**Fig 4:**
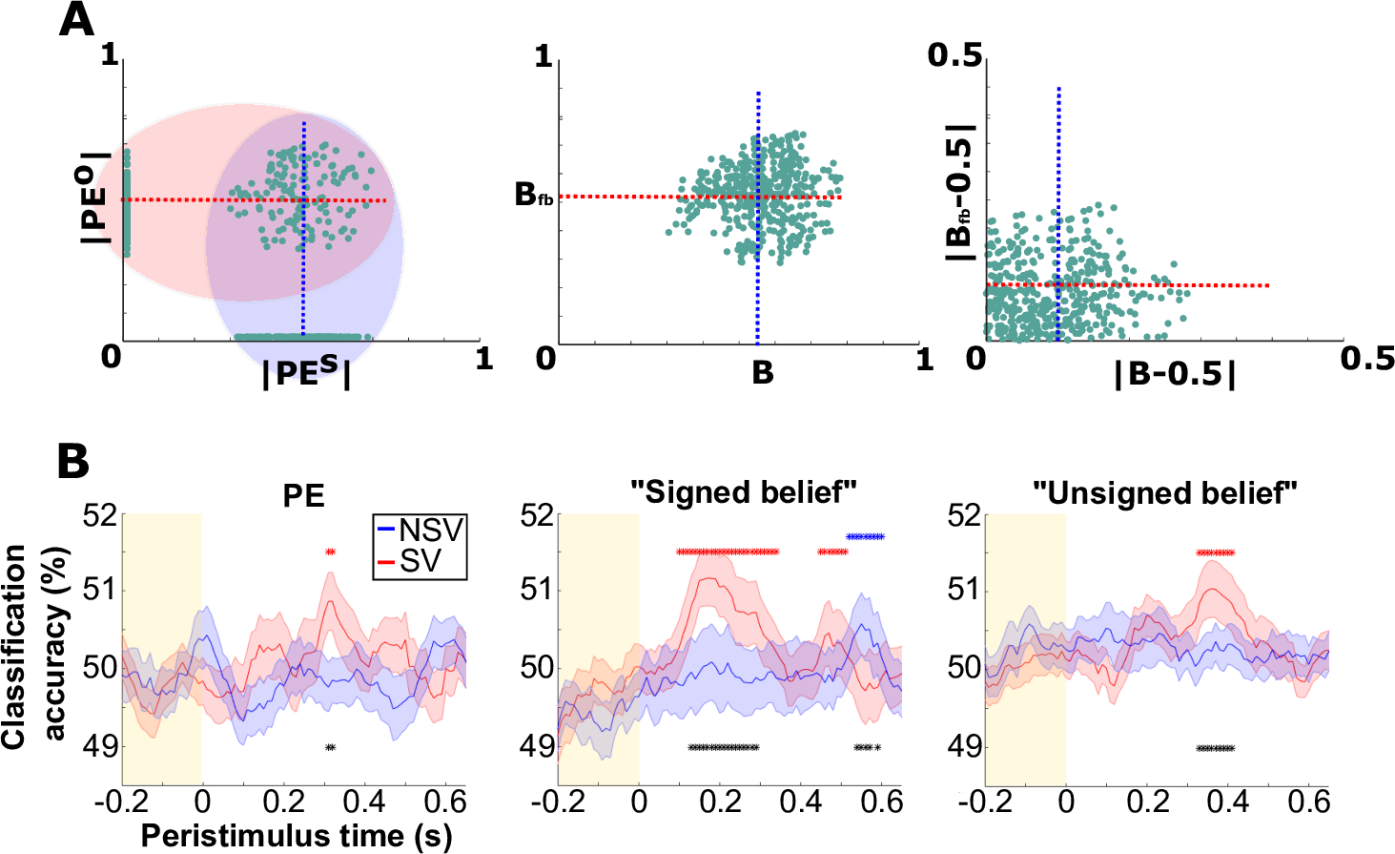
Decoding agent identity from the neural encoding pattern of learning signals. (A) Generation of pseudotrial data. These plots show the distributions of, and relationship between, trial-wise variables for the 2 agents (self on x-axis and other on y-axis). The left panel is for the |PE| variable, the middle panel is for the ‘signed belief’ variable, and the right panel is for the ‘unsigned belief’ variable. These are the typical estimates for 1 subject, and each dot corresponds to a sampling trial. In all 3 plots, the vertical blue line shows the median split used to generate pseudotrials that were labelled as ‘self’, while the horizontal red line shows the median split used to generated pseudotrials that were labelled as ‘other’. Note that in the left panel, the data form 3 clusters. The cluster of points on the x-axis correspond to ‘privileged’ trials, the cluster of points on the y-axis correspond to ‘decoy’ trials, and the cluster of points in the middle correspond to ‘shared’ trials. In order to generate the |PE| pseudotrials, the median splits were only performed on data for which the |PE| was nonzero. (B) Time course of group mean CAs in the SV (red) and NSV (blue) for the |PE| pseudotrials (left), ‘signed belief’ pseudotrials (middle), and ‘unsigned belief’ pseudotrials (right). Classifiers trained on all 3 types of pseudotrials could predict agent identity in the SV (*P* < 0.05 FWE-corrected). However, in the NSV, the classifiers only showed a small, late decoding effect for the ‘signed belief’ pseudotrials. In all 3 analyses, there were multiple time points in which CA was significantly higher in the SV than in the NSV (*P* < 0.05 FWE-corrected). Red stars indicate significant above-chance classification for the SV. Blue stars indicate significant above-chance classification for the NSV. Black stars indicate significant above-chance difference between the SV CA and the NSV CA. Shaded regions show SEM across subjects. See S7 Fig for spatial mapping of decoding accuracy. See S6 Fig for neural decoding of visual stimuli. See S1 Data for all numerical values. B, belief about the Bernoulli parameter; B_fb_, belief about the other agent’s false belief about the Bernoulli parameter; CA, classification accuracy; FWE, fair-wise error; NSV, nonsocial version; PE, prediction error; PE^o^, other-attributed prediction error; PE^s^, self-attributed prediction error; SV, social version.

We wanted to test whether we could distinguish a neural pattern encoding a PE^s^ from a pattern encoding a PE^o^ and thus determine whether a self–other distinction can be achieved on the basis of these signals. A typical way to identify the neural pattern encoding a PE is to regress the magnitude of the PE (derived from our learning model) against the brain activity, across trials. This would yield a single beta estimate at each sensor, capturing the slope of the relationship between PE and brain activity at that sensor. However, in order to use powerful multivariable methods like support vector machine (SVM) classification to look for differences in the spatial patterns of PE^s^ and PE^o^, it was necessary to obtain multiple samples of each pattern. One way to achieve this is to divide the data into multiple partitions (without replacement) and repeat the analysis in each partition to obtain multiple independent samples of the spatial pattern for each type of PE. This is the approach we opted for, using the smallest possible partitions: pairs of trials (Fig 4A).

To maximise power without introducing bias, we randomly partitioned trials into pairs under the constraint that each pair contained 1 trial above the median |PE| and 1 trial below the median |PE|. Thus, the difference in brain activity between the 2 trials within a pair corresponded to a representation of |PE|. We performed this random partitioning independently for PE^s^ and PE^o^. This resulted in 2 sets of difference images, corresponding to neural representations of |PE^s^| and |PE^o^|. Finally, we could then apply multivariable methods to classify whether each difference image was a representation of |PE^s^| or |PE^o^|.

It should be noted that this method differs slightly from typical pattern-based neuroimaging analyses described in, for example, [33]. Usually, such an analysis looks for a neural representation of some variable. This is achieved by training a classification or regression model to distinguish patterns of neural activity corresponding to different values of that variable. Above-chance accuracy of the model indicates that the brain activity contains information about the variable. However, in our case, we were interested in a difference in the representation of a variable between 2 conditions. Because the representation itself is defined by a difference in neural activity between a large PE and a small PE, we were looking for a difference of differences. Thus, it was necessary to train classifiers on patterns of subtracted activity rather than activity patterns from individual trials.

We started with *N* trials in total. First, we partitioned all ‘privileged’ and ‘shared’ trials (2*N*/3 trials) by median split on |PE^s^|. We then randomly sampled 2 trials, one from either side of this partition, and subtracted the ERF on the low |PE^s^| trial from the ERF on the high |PE^s^| trial, at every sensor and time point. For ease of reference, we call this contrast image a ‘pseudotrial’. We continued randomly sampling pairs of trials without replacement to obtain a total of *N*/3 pseudotrials. Each of these pseudotrials describes the difference in activity between a trial with a high |PE^s^| and a trial with a low |PE^s^|. The brain activity in the difference image thus constituted a representation of |PE^s^|.

Second, we partitioned all ‘decoy’ and ‘shared’ trials (2*N*/3 trials) by median split on |PE^o^|. We carried out the same procedure as for PE^s^, resulting in a second set of *N*/3 pseudotrials, each of which constituted a representation of |PE^o^|. At each time point, we trained a classifier to distinguish PE^s^ pseudotrials from PE^o^ pseudotrials.

We tested classifiers in crossvalidation, yielding a time course of classification accuracies (CAs). The absolute difference in CA underlying reliable effects was, in some cases, as small as 1%. In observing this, we note that effect sizes cannot be inferred from absolute CAs [34–36]. Therefore, to make statistical inferences, we adopted a permutation-based method to determine whether any CA was significantly better than chance. This procedure has been recommended for making inferences on ‘information-based’ neural measures such as CA [35].

To derive a threshold for statistical significance, we repeated the whole pseudotrial analysis many times, each time using data generated from a permuted trial sequence. For every permutation, we took the maximal CA (or maximal difference in CA between the SV and NSV) across all time points. We thus generated a null distribution of maximal CAs (or maximal CA differences). The 95th percentile of the distribution was taken as our threshold for statistical significance. This procedure allowed us to make statistical inferences without making assumptions about how CAs (or CA differences) are distributed, whilst also correcting for multiple comparisons across time points, at a 0.05 FWE level.

We found that self and other could be classified significantly above chance level from the spatial patterns of activity that represented |PE^s^| and |PE^o^| approximately 300 ms after stimulus onset (Fig 4B). However, CA did not exceed chance level when we conducted this same analysis on the NSV data. Moreover, at approximately 300 ms, there was a significant difference between CA in the SV and CA in the NSV. Thus, distinct spatial activity patterns for |PE^s^| and |PE^o^| were evident in the SV but not in the NSV. This implies information about self and other is intrinsic to the representations of low-level learning signals, whilst information about self and counterfactual self is not.

To test the robustness of this finding, we performed 2 additional variants of the analysis, by constructing pseudotrials from subjects’ trial-wise ‘signed beliefs’ (B and B_fb_) and ‘unsigned beliefs’ (|B − 0.5| and |B_fb_ − 0.5|). The former are the subject’s trial-wise estimates of the underlying Bernoulli parameter from the perspective of each agent. The latter are the absolute distances of these estimates from 0.5, which represents an equal probability of either outcome. The ‘unsigned belief’ is thus a measure of confidence in what the next outcome will be. It should be noted that here, we can use all *N* trials to generate pseudotrials. Thus, we end up with *N*/2 pseudotrials for each class and *N* pseudotrials in total. We found that classifiers trained on pseudotrial data, generated from either of these latent variables, could predict agent identity (self or other) significantly above chance in the SV. However, in the NSV, the classifiers could only predict agent identity (self or counterfactual self) for pseudotrials generated from ‘signed beliefs’, and in this instance, the signal was weaker and occurred later in time than was the case for the SV (Fig 4B). Furthermore, we found that CAs for the SV were significantly larger than CAs for the NSV at multiple time points for both of these pseudotrials. Finally, for comparison, in a separate analysis classifying between the visual stimuli, we obtained similar decoding accuracies in the SV and NSV (S6 Fig).

### Neural decoding of agent identity predicts self–other distinction

An important question is whether the neural distinction in learning signals is related to a behavioural measure of self–other distinction. A subset of our behavioural models (models 11 to 19) included a ‘leak’ (λ) parameter that governed the extent to which PE^s^ was erroneously used to update B_fb_ and/or PE^o^ was erroneously used to update B, thus indexing an inability to discriminate between 2 different agents’ learning processes. We estimated λ values by selecting the best-fitting λ-containing model for each individual subject. If the best model contained 2 λ parameters, we took the mean of the 2 values. We derived 2 estimates of λ for each subject, one for the SV and one for the NSV.

We then computed, for each subject, a metric describing overall neural self–other distinction. In order to do this, we took the maximal CA from each of the time courses from the 3 types of pseudotrial (Fig 4B) and summed these 3 numbers. This provided one number for neural agent decoding in the SV and another number for neural agent decoding in the NSV.

**Fig 5:**
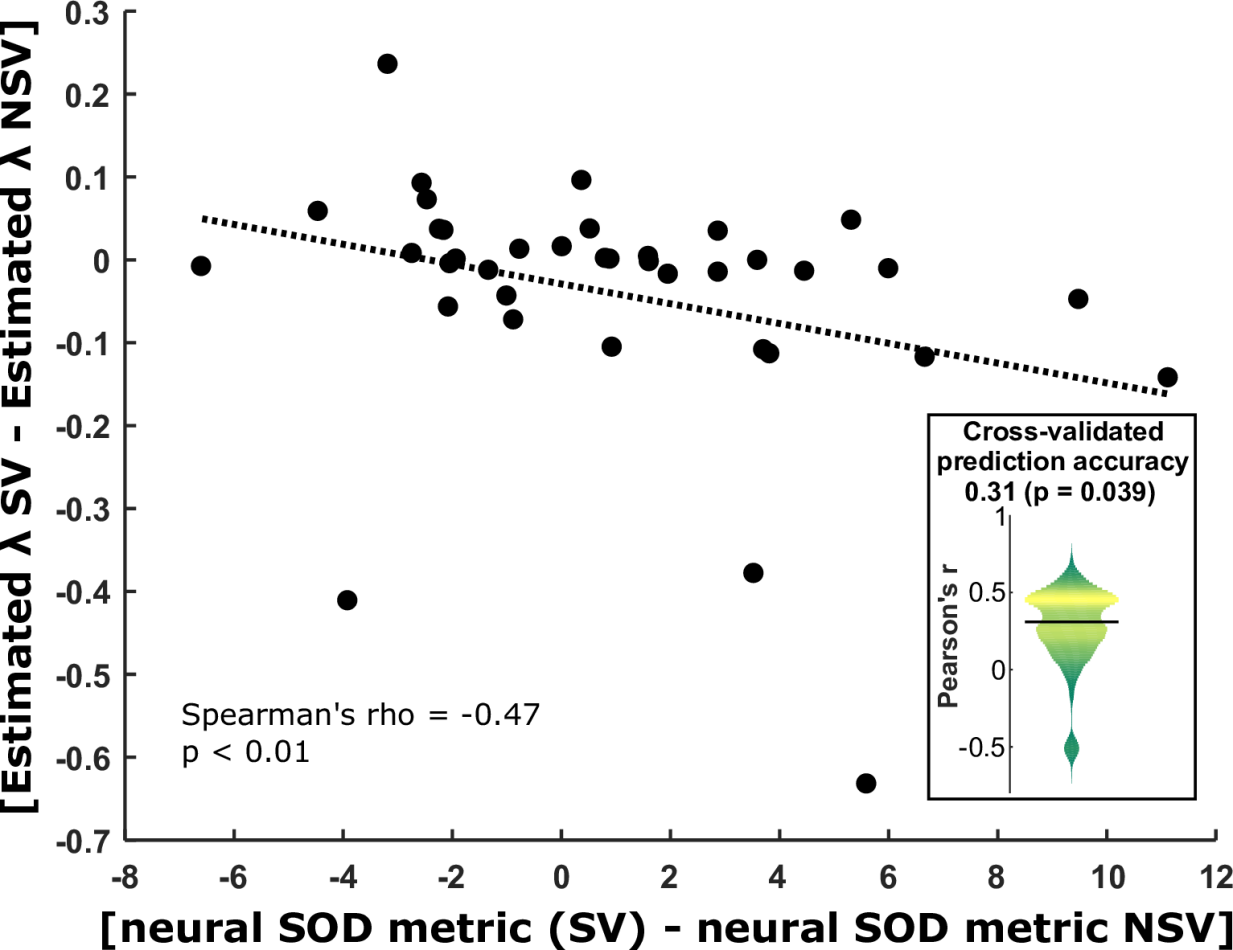
Relationship between neural agent decoding and behavioural agent distinction. Each dot is a subject. The x-axis shows the contrast (SV − NSV) of maximum CAs, pooling data from all 3 types of pseudotrial. The y-axis shows the contrast (SV − NSV) of estimated λ parameters, which quantify misattributing belief updates to the wrong agent’s belief. The negative correlation implies that the subjects for whom we obtained more accurate agent decoding in the SV than the NSV also behaviourally better discriminated between agents in the SV than the NSV. The inset violin plot shows the distribution of prediction accuracies (correlation coefficients) of a linear regression model across 10,000 folds of crossvalidation, in which neural SOD (SV − NSV) was used to predict estimated λ (SV − NSV). The horizontal black bar indicates the median of this distribution. See S1 Data for all numerical values. CA, classification accuracy; NSV, nonsocial version; SOD, self–other distinction; SV, social version.

Because λ in the SV and λ in the NSV were strongly correlated across subjects, we examined the difference between the SV and NSV. Due to the non-normally distributed parameter estimates, we computed a nonparametric Spearman’s rank correlation coefficient. We found a strong negative correlation (Fig 5) between the neural decoding contrast (SV − NSV) and the estimated λ contrast (SV − NSV): Spearman’s rho: −0.43, *P* < 0.01. We also tested the accuracy of a linear regression model that used neural decoding contrasts to predict the estimated λ contrasts. Here, we used crossvalidation with random subsampling (train on half, test on half) and recorded the correlation between predicted and observed values on every fold. The median Pearson coefficient across 10,000 folds was 0.31, which was significantly greater than chance as determined by a nonparametric permutation test (*P* = 0.039).

We also found a significant positive correlation (Spearman’s rho: 0.32, *P* < 0.05) when, instead of using raw parameter estimates, we used the relative model evidence (BIC) of a subject’s best lambda-containing model and best nonlambda model. In other words, subjects whose SV behaviour is better explained by a model with lambda parameters than their NSV behaviour show less neural self–other distinction in the SV than in the NSV.

These findings show that, in subjects for whom agent identity could be more accurately decoded in the SV than in the NSV, there was also more behavioural evidence for segregating the beliefs of self and other in the SV than in the NSV. This suggests that the distinctiveness of neural patterns encoding learning signals attributed to 2 different agents is predictive of how well a subject behaviourally succeeds in distinguishing between these 2 agents’ beliefs.

### Neural decoding of agent identity predicts subclinical psychopathological traits

Finally, we asked whether neural agent decoding relates to intersubject differences in subclinical personality traits. All subjects filled out 5 questionnaires of interest: Beck Depression Inventory (BDI), Empathy Quotient (EQ), Interpersonal Reactivity Index (IRI), Inventory of Callous-Unemotional traits (ICU), and the Community Assessment of Psychic Experience (CAPE). These questionnaires were specifically chosen to assess the presence of psychopathological traits previously proposed to relate to a dysfunctional self–other distinction or more general social cognitive deficits [17, 21, 22, 25]. These questionnaires assessed autistic (EQ), schizotypal (CAPE), antisocial (ICU), and depressive (BDI) traits as well as general capacities for empathy and sympathy (EQ, IRI). We also obtained measures of response bias using an additional questionnaire, the Balanced Inventory of Desirable Responding (BIDR) [37]. None of the subjects were considered to have an unacceptably high response bias (see Materials and methods).

**Fig 6:**
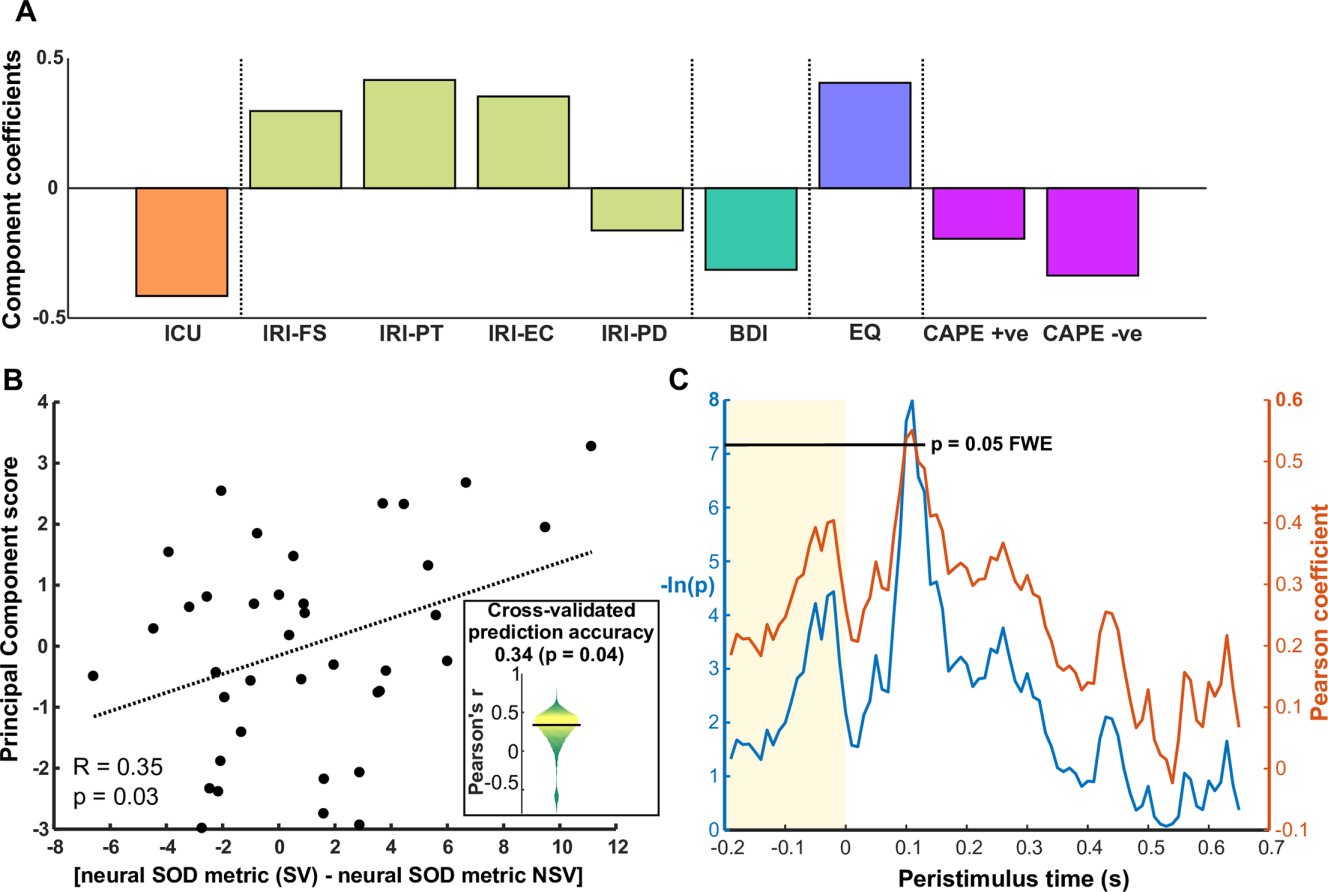
Relationship between neural agent decoding and personality traits. (A) Component coefficients (loadings) for personality data in the PC1. This component loads negatively on psychopathological traits, including both subscales of the CAPE questionnaire (schizophrenia), the BDI (depression), the personal distress subscale of the IRI, and the ICU (unemotional and callous traits). (B) Correlation between neural SOD metric (SV − NSV) with PC1 scores. Each dot is subject. The inset violin plot shows the distribution of prediction accuracies (correlation coefficients) of a linear regression model across 10,000 folds of crossvalidation, where neural SOD (SV − NSV) was used to predict PC1 scores. The horizontal black bar indicates the median of this distribution. (C) Pearson coefficient time course (red) from correlating subjects’ PC1 scores with (SV CA − NSV CA) for the classifiers trained on ‘signed belief’ pseudotrials. A time course of corresponding −ln(p) values is shown (blue) to indicate the significance of the correlation at each time point. The horizontal black line shows the permutation-based corrected threshold for statistical significance in terms of −ln(p). See S1 Data for all numerical values. +ve, positive symptoms; −ve, negative symptoms; BDI, Beck Depression Inventory; CA, classification accuracy; CAPE, Community Assessment of Psychic Experience; EC, empathic concern; EQ, Empathy Quotient; FS, fantasising; ICU, Inventory of Callous-Unemotional traits; IRI, Interpersonal Reactivity Index; NSV, nonsocial version; PC1, first principal component; PD, personal distress; PT, perspective taking; SOD, self–other distinction; SV, social version.

We performed dimensionality reduction on age and gender-controlled personality questionnaire data (see Materials and methods) using a principal components analysis (PCA), having included all subscales of the 5 questionnaires of interest, giving 9 dimensions in total (Fig 6A). The first principal component (PC1) explained 32% of the variance in the questionnaire data and loaded negatively with both subscales of the CAPE questionnaire (schizophrenia), BDI (depression), ICU (antisocial behaviour), and 1 subscale of the IRI (personal distress in social situations); it also loaded positively with EQ and other subscales of the IRI (Fig 6A). Thus, the PC1 negatively captured psychopathological features in our personality data in a nonspecific manner. We projected the personality data into the space of this principal component to obtain a score for each subject.

First, we correlated the neural self–other distinction metric, as described in the previous section, with the PC1 scores. When using the contrast of (SV − NSV), this yielded a significant correlation: R = 0.39, *P* = 0.017 (Fig 6B). We also tested the accuracy of a linear regression model that used neural decoding contrasts to predict the PC1 scores, using the same method as described for Fig 5. The median Pearson coefficient across 10,000 folds was 0.34, which was significantly greater than chance as determined by a nonparametric permutation test (*P* = 0.04). Therefore, subjects for whom we obtained higher CAs in the SV than in the NSV scored higher on the PC1. In other words, subjects for whom it was easier to neurally decode self from other than to decode self from counterfactual self scored higher on a nonspecific anti-psychopathological component. When looking at the SV and NSV neural self–other distinction metrics separately, we found a significant positive correlation for the SV (R = 0.43, *P* < 0.01) but no significant correlation for the NSV (R = 0.01, *P* = 0.94).

We then investigated the temporal evolution of this relationship for each of the 3 types of pseudotrial. At each peristimulus time sample, we correlated the subjects’ PC1 scores with (CA SV − CA NSV) to generate a time course of Pearson coefficients (Fig 6C). Using permutation-based thresholding, to correct for multiple comparisons in time and between the 3 types of pseudotrial, we found a significant positive correlation (*P* < 0.05 FWE) approximately 110 ms after stimulus onset (Fig 6C) when using the ‘signed belief’ pseudotrials. This falls within the window of significant self–other distinction in signed beliefs (100–340 ms) as shown in Fig 4B.

## Discussion

We show that a representation of a learning signal (PE or belief) is encoded with a different neural spatial pattern when the signal is attributed to self as compared to when it is attributed to another agent. Intersubject variability in this difference correlated between subjects with a behavioural measure of self–other distinction and with subclinical psychopathological traits. This suggests that self–other distinction is realised by an encoding of agent identity that is intrinsic to low-level learning signals, and the fidelity with which this occurs is an important dimension of variation between individuals.

In our experiment, subjects had to solve 2 simultaneous computational problems. The first problem was predicting what the next outcome would be. The second problem was identifying whether this belief-state about the next outcome should be attributed to one agent or another, a computation that requires a self–other distinction. We found a spatial segregation between self-attributed and other-attributed learning signals. This means that the neural representations of beliefs and PEs in this task also contained information about the agent to whom these signals belong, and consequently, the neural resources that compute the next outcome also inevitably contribute to computing a self–other distinction. It is of interest, therefore, that the degree of spatial segregation was correlated with a behavioural measure of self–other distinction derived from our learning models.

Previous work has shown that neuronal populations in the macaque anterior cingulate cortex preferentially encode simulated RPEs [10] and the future decisions [38] of another monkey. Conversely, human fMRI data has identified common activations in the mPFC that represent RPEs [6] or subjective preferences [12] for both self and other in an agent-independent manner. Likewise, mirror neurons recorded from the macaque premotor cortex are also agent independent [2]. A self–other distinction in the affective domain has been reported in terms of dissociable networks for experienced versus vicarious pain [39, 40], though other reports suggest that these are both subserved by the same structures [41, 42].

The above accounts are conflicting with respect to whether self- and other- attributed signals share common or distinct neural activations. One possible reason for this is that previous studies eliciting simulated signals were not indexing a self–other contrast per se but rather a contrast of executed behaviour versus observed behaviour, in which the subject receives feedback from the observee’s behaviour. In these cases, learning or decision variables are discriminated not by virtue of the agent to whom they are attributed but instead by virtue of distinct input modalities and cognitive demands required for instrumental learning and observational learning, respectively. Because these factors are heavily task specific—for instance, dependent on the way in which the subject accrues information about the other agent’s behaviour—it is unsurprising that observational learning paradigms have produced inconsistent accounts of the neural encoding of agent identity.

In the present study, we devised a self–other contrast per se by allowing subjects to observe what the other agent observed but not the other agent’s behaviour. By requiring subjects to switch between attributing a signal to self and attributing a signal to other, with fixed sensory input modalities and cognitive demands, we could show that learning signals do contain information about the identity of the agent to whom they are attributed. Consistent with this are our findings that the neural self–other distinction is modulated by individual personality traits, as well as by the precise contextual relationship between the agents in question, as assessed with the SV and NSV of the paradigm. Our findings do not rule out a possibility that the brain uses additional mechanisms to distinguish self from other, for instance, with an explicit encoding of agent identity that is separate from low-level learning signals. However, our results support the theory that agent-specific learning signals are sufficient for the brain to achieve a self–other distinction during mentalising.

Our results support the idea that the brain updates simulated beliefs of another agent using PEs calculated within the frame of reference of that agent. Previous work on other-referenced processing has shown that humans and other primates simulate another agent’s experience of unexpected reward [3, 6, 10]. Our work extends these findings to the domain of updating beliefs about non–reward-related quantities. A simulated sensory PE such as what we observed, combined with information about a preceding state, could also be used to simulate how another agent learns transition models of complex environments with multiple states, a requisite for goal-directed behaviour [43–47].

Differences between neural representations of signals attributed to self and other, which we attribute to agent identity, might relate to some other features of the task frame [48–50]. However, if this were true, we would expect representations to be similarly distinct in the NSV as in the SV because both versions shared a shift in task frame (between self and other in the SV and between self and a counterfactual self in the NSV). The finding that representations were significantly less distinct in the NSV supports our conclusion that features intrinsic to agent identity are fundamental to the distinct representations observed in the SV. Although representations were overall less distinct in the NSV, there was nevertheless a detectable difference in ‘signed belief’ representation between self and counterfactual self, occurring approximately 550 ms after stimulus onset. It will be important in future work to clarify why this long latency separation occurs in a nonsocial context.

The differences we found between the SV and NSV do not necessarily mean that subjects were not engaging in social computations in the NSV. Despite evidence for a so-called Theory of Mind network [51–53] recruited during mentalising, there is evidence that the brain might also rely on domain general computations for social cognition [54, 55]. It has been suggested that ‘social’ computations like mental state inference and ‘nonsocial’ computations, such as mental time travel and metacognition, are underpinned by the same general capacity for metarepresentation [25, 56–58]. If self–other distinction is a special case of a domain-general computation, future work should seek to understand why this computation is executed differently in social and nonsocial contexts.

We observed substantial intersubject heterogeneity in the spatiotemporal pattern of PE signals in our task. Although anatomical inferences are limited for data acquired in sensor space [59, 60], the heterogeneity would suggest a diversity of cortical regions encoding PEs. fMRI studies, employing both learning and nonlearning paradigms, have reported unsigned sensory PE activity or activity corresponding to unexpected neutral stimuli in a range of cortical and subcortical regions, including the anterior insula and inferior frontal gyrus [61], primary sensory cortices [62–64], superior temporal sulcus [65], hippocampus [66], cerebellum [67], striatum [64, 68, 69] and midbrain [70].

With regards to timing, previous studies in a nonsocial context using electroencephalography (EEG) [71–73] and MEG [74] have identified signed PE signals 200 to 350 ms after stimulus onset. However, unsigned PE signals are less well characterised and appear to be encoded across a much broader time window, ranging from 145 ms to 640 ms after stimulus onset [71]. Finally, previous false belief experiments using EEG [75, 76] and MEG [77] have reported latencies ranging from 100 ms to 800 ms after stimulus onset, at which time signals have differentiated true beliefs from false beliefs. Here, we show that in a social setting, these PE signals are agent specific as soon as they are detectable, approximately 300 ms after stimulus onset. Conversely, in the NSV, these PE signals were not agent specific at any time point.

The contrast in agent decoding accuracy between the SV and NSV correlated with subjects’ behavioural ability to differentiate between agents and with the PC1 of subclinical personality traits. Specifically, subjects for whom self and other brain representations were more distinct than self and counterfactual self scored higher on this principal component. An inability to differentiate between self and other is a feature of psychopathology [17–24]. Our measures of agent decoding might be useful as a sensitive gauge of a self–other distinction in the context of phenotypic markers for psychopathology.

Computational phenotyping in psychiatry has recently been mooted [78] and posits individualised diagnostic and therapeutic tools in mental health based on computational models of behaviour and brain function. Recent efforts to develop computational models of Theory of Mind [79, 80] do not address how representations are attributed to different agents. Here, we present the foundations for a model of Theory of Mind that specifically addresses computations that contribute to a self–other distinction, a quantifiable characteristic necessary in both social and nonsocial contexts.

## Materials and methods

### Ethics statement

Ethical approval for this study was obtained from the University College London Research Ethics Committee, application number 9929/002.

### Participant details

Forty-one healthy adults (23 female) aged 18 to 42 participated in the experiment. They were recruited from the UCL Institute of Cognitive Neuroscience subject pool. All participants had normal or corrected-to-normal vision and had no history of psychiatric or neurological disorders. One participant was excluded from the analysis due to excessive head movements in the scanner and an additional 2 were excluded due to technical faults with MEG data acquisition, leaving 38 subjects (21 female) for the analysis, with a mean age of 26.6 (SD 6.9). All participants provided written informed consent.

### Pipeline for generating trial sequences

We followed a stringent pipeline to minimise the correlation between our variables of interest. First, we generated 2 random walks to represent the time courses of P and P_fb_. One walk started with a value randomly selected from a uniform distribution bound between 0.1 and 0.3, and the other walk started with a value randomly selected from a uniform distribution bound between 0.7 and 0.9. The walks proceeded with step sizes of 0.025. The sign of this step was random in most instances, but because P and P_fb_ are probabilities, the 2 walks were bound between 0 and 1. To achieve this, the walks were always reflected by these boundaries, which sometimes required a reversal of the randomly selected step sign. The walks were terminated after 408 steps. This resulted in 2 pseudorandom walks, each with 408 data points. If these 2 datasets had a nonsignificant Pearson correlation coefficient, they were saved. This process was repeated iteratively until 300 pairs of uncorrelated pseudorandom walks had been generated. Each pair of walks was then used to generate a trial sequence.

To generate the trial sequence, we first generated a sequence of 408 trial types (‘privileged’, ‘shared’, or ‘decoy’). This sequence consisted of 34 concatenated blocks of 12 trials, such that each block was a random sequence of 4 of each trial type. Thus, trial type frequencies were balanced throughout the experiment. We then used this sequence of trial types along with one of the pairs of random walks to simulate a sequence of Bernoulli trials. One of the random walks represented P and the other represented P_fb_. For a trial t_i_, if the trial type was ‘privileged’, we drew from a Bernoulli distribution with P equal to the i^th^ data point in random walk P. If the trial type was ‘decoy’, we drew from a Bernoulli distribution with P equal to the i^th^ data point in random walk P_fb_. If the trial type was ‘shared’, we drew from a Bernoulli distribution with P equal to 0.5. This resulted in 1 sequence of 408 ‘heads’ and ‘tails’. The complete trial sequence consisted of a sequence of Bernoulli outcomes and a corresponding sequence of trial types.

We then simulated an agent that observed this trial sequence to generate trial-wise estimates of our variables of interest: B, B_fb_, PE^s^, and PE^o^. In order to do this, we used the 2-parameter RW model (model 3 in Table 1), which was the simplest RW model in our model space. We selected parameters by taking the mean of parameter estimates across 18 subjects in a separate pilot study; we used a learning rate (α) of 0.1. We tested for correlations between B and B_fb_ and between |PE^s^| and |PE^o^|. If these correlations were nonsignificant, we saved the trial sequence and moved on to the next pair of random walks. It should be noted that on ‘decoy’ trials, PE^s^ was coded as 0 while PE^o^ was usually nonzero, but on ‘privileged’ trials, PE^o^ was coded as 0 while PE^s^ was usually nonzero (Eq. 1). This meant that there was always a negative correlation between |PE^s^| and |PE^o^| (Fig 4A). Therefore, we tested for correlations between these regressors only on ‘shared’ trials, in which both values were nonzero. If a significant Pearson coefficient was discovered, the process was attempted again with a different pseudorandom sequence of trial types. If after 300 iterations no trial sequence was generated that provided uncorrelated variables, we moved on to the next pair of random walks. At the end of this process, we ended up with 158 suitable trial sequences.

### Cover stories and task details

Every subject played 2 versions of this game while inside the MEG scanner—an SV and an NSV—one after the other. We created 2 cover stories that could interchangeably be applied to either the SV or NSV, allowing for 4 possible games to be played (SV1, SV2, NSV1, or NSV2). Our cover stories were designed to be immersive and to make the underlying structure of the task, particularly the drifting Bernoulli parameter (P), as intuitive as possible. Each subject played 2 games with different cover stories to make the SV and NSV feel as different as possible. Each subject was allocated to 1 of 4 groups. These groups were defined by the order in which the SV and NSV were played and the cover stories that were applied to both of them. Group 1 played SV1 → NSV2. Group 2 played NSV2→ SV1. Group 3 played SV2 →NSV1. Group 4 played NSV1 → SV2. This 2 × 2 factorial design meant that game order and cover story mappings were counterbalanced across subjects. For each subject, 2 of the 158 pregenerated trial sequences were selected, 1 for their first game and 1 for their second game.

In cover story 1, subjects played the role of a ‘shop assistant’ working in a shop on a tropical island selling only pink umbrellas and yellow sun-shades (i.e., ‘heads’ or ‘tails’). On every trial, a ‘customer’ would come to the shop and buy an umbrella or a sun-shade. The weather on the island was unknown, but it was always changing, and this was reflected in the items that the customers chose to buy. It was the job of the shop assistant to observe and remember the sequence of sales to infer the gradual changes in the weather in order to make predictions about what the next customers would buy. In cover story 2, subjects played the role of an assistant in a shop in the centre of a city selling only red cans of cherry-cola and blue cans of diet-cola. Outside the shop there were large digital advertisements, which were always showing images of these products. The advertisements could be biased towards one of the products, but they were always changing. The assistant could not see the advertisements. On every trial, a customer would come to the shop and buy one of the drinks, as determined by which product was currently favoured by the advertisements outside. It was the job of the shop assistant to observe and remember the sequence of sales to infer the gradual changes in the advertisements in order to make predictions about what the next customers would buy. In both cover stories, subjects were instructed that the hidden states (weather or advertisements) change constantly and slowly and that they must consider every sale in order to keep track of the fluctuations. These environmental fluctuations provided cover stories to justify the random drifting of P.

The differences between the SV and NSV applied to both cover stories. In the SV, there was an accompanying ‘manager’. The manager represented a real person. This was another participant, outside the scanner in a different room. Subjects fully understood the perspective of this other person because they had previously participated in that role (see section Full protocol). Subjects were told that the manager spends some time in a ‘back room’ and therefore does not get to observe all of the sales. Therefore, some of the sales contained ‘privileged’ information that only the shop assistant could see. However, on some trials, the manager came out of the ‘back room’ and did get to see the sale. These were the trials with ‘shared’ information. Finally, subjects were told that the manager was watching ‘CCTV footage’ from the ‘back room’ to keep track of the sales but that the manager was unaware that this was actually last week’s footage, so the information was misleading. The shop assistant was provided with a video link of what the manager was watching on closed-circuit television (CCTV) so they could see all the misleading information that the manager was receiving (‘decoy’ trials). Subjects had to try and keep track of the ‘manager’s’ beliefs.

In the NSV, there was a ‘hi-tech cash register’ instead of a manager. Instead of a real person’s beliefs, subjects had to keep track of a computer’s belief-state. Subjects were told that the cash register keeps a record of every sale that’s been entered to it and computes a prediction of what the next customer will buy. Subjects were also told that some customers buy with coupons instead of cash and that these sales would not be entered into the cash register. These were the ‘privileged’ trials. Subjects were told that some customers pay with cash and in these instances, the cash register would be used and it would update its prediction. These were the ‘shared’ trials. Finally, subjects were told that the cash register had an internet connection to a partner shop and received updates from the sales happening there. However, the partner shop was far away from this shop, on another island with different weather for cover story 1, or in another city with different digital advertisements for cover story 2. When these sales occurred, the ‘cash register’ was updated with misleading information, and the subject could also see this information (‘decoy’ trials).

All 4 games had identical designs and trial structures (Fig 1A). A sampling trial was one in which the subject samples the environment on behalf of themselves (‘privileged’), on behalf of the manager/cash register (‘decoy’), or on behalf of themselves and the manager/cash register at the same time (‘shared’). Sampling trials started with cue image (Fig 1B) on the screen to indicate whether it was going to be a ‘privileged’, ‘decoy’, or ‘shared’ trial. The cue was presented in the centre of the screen with a grey background for 1,100 ms. Then, the cue disappeared, and an outcome was presented (Fig 1C) at the centre of the screen for 900 ms. The outcome represented what the current customer had chosen to buy (i.e., the outcome of the Bernoulli trial: heads or tails). Then, the outcome disappeared and was followed by an intertrial interval (ITI) with a central fixation cross that lasted 750 to 1,250 ms.

After 4 to 9 sampling trials, there was a probe trial (Fig 1A) in which subjects reported their estimate of either P or P_fb_. Subjects were told that the 2 products of their shop were kept in 2 separate boxes. On probe trials, a horizontal scale appeared on the screen with one of the boxes on the left and the other box on the right. The positions of the 2 boxes were randomly generated on every probe trial, to avoid any directional biases. A probe trial could be a ‘self’ trial (report P) or an ‘other’ trial (report P_fb_). On ‘self’ trials, subjects were probed with the question ‘Which box would YOU reach into now?’ as if anticipating what the next customer would buy. Subjects had 7 seconds to give their response by moving a virtual arrow left or right along the scale and then pressing an ‘enter’ button once they were happy with the position. The arrow was initially invisible but appeared in a random location along the scale as soon as subjects pressed left or right. This was to avoid any systematic biases that the starting location of the arrow might have induced. ‘Other’ trials were different between the SV and NSV. In the SV ‘other’ trials, subjects were asked ‘Which box would the MANAGER reach into now?’ and subjects had to respond by putting themselves into the shoes of the manager and reporting their estimate of P_fb_. In the NSV, this question was rephrased as ‘Which box would you reach into now IF you used the readout on the cash register?’ Subjects never received any feedback from their choices on probe trials, so they never knew how accurate their responses were. Probe trials were randomly selected to be ‘self’ or ‘other’, so subjects never knew which question was going to come next and they had to try to keep track of P and P_fb_ at all times. However, subjects were never probed with the same question more than 3 times in a row.

Subjects were incentivised to perform well because their final payment at the end of the experiment depended on their accuracy scores on probe trials. We calculated scores based on how much subjects’ responses deviated from the ‘optimal’ response on any given probe trial. In the SV ‘other’ trials, the ‘optimal’ response was whatever the other participant selected for that trial as the ‘manager’. For all other probe trials, the ‘optimal’ response was taken from the random walks P or P_fb_. Once again, participants never got to see these scores nor received any kind of feedback until they were paid at the end of the experiment.

Before playing the games, participants were carefully instructed and well practiced to make sure they understood that after a probe trial, the hidden state of the environment would continue from the state it was in before the probe trial. Specifically, they were instructed that after a probe trial, they should not treat the upcoming information as independent of what they had already seen but rather treat the entire stream of sampling trials as a continuous sequence while the hidden state of the environment (weather or advertisements) changed gradually.

### Full protocol

Deception was never used in this experiment; the setup was exactly as it was described to participants. All participants came to the lab on 2 occasions, T1 and T2. T2 occurred no more than 4 days after T1. We will explain both sessions with the fictional participants ‘Sally’ and ‘Anne’. Sally and Anne were both in group 1, which meant that while they were in the scanner, they played SV1 followed by NSV2, but they never played SV2 or NSV1. Anne arrived at the lab at time T1 for a behavioural session with no scanning. Anne was instructed how to play a simplified version of SV1 called simpleSV1. In simpleSV1, Anne played the role of the ‘manager’. The game proceeded exactly as SV1 (described above) but with the following differences: (1) there was no mention of a shop assistant; (2) ‘privileged’ trials were excluded; (3) Anne was instructed to ignore the cue images and told that they were not relevant to the game; and (4) there was only one type of probe trial, which asked ‘Which box would YOU reach into now?’ The presence of cue images was justified with instructions that said that the images simply showed whether Anne (as the ‘manager’) was seeing the sale directly or via CCTV from the back room but that this was irrelevant for Anne’s task.

After this, Anne was instructed how to play a simplified version of NSV2 called simpleNSV2. In this version of the game, Anne played the role of a ‘shop assistant using the information from a hi-tech cash register’. The same differences applied as in simpleSV1. The presence of cue images was justified with instructions that said that the images simply showed whether the cash register was getting information from a sale in this shop or in a partner shop next door but that this was irrelevant for Anne’s task. We did not analyse the behavioural data from simpleSV1 or simpleNSV2.

Finally, Anne was given the following set of personality questionnaires to complete: BIDR, EQ, IRI, CAPE, ICU, and BDI. Anne and all other participants filled out the BIDR first in order to provide us with a measure of response bias. The subsequent 5 questionnaires were completed in a random order.

The next day, Anne returned for session T2. She was then informed that there was a social element to the experiment and that another participant, Sally, was also there but that Sally was doing a T1 session—i.e., Sally was doing what Anne had done the previous day. We walked Anne past a testing room so that she would briefly see Sally to convince her that there was indeed another participant. Anne was then taken to a different testing room where she played a short working memory (n-back) task and then learned how to play SV1 and NSV2 with extensive practice. We explained to Anne that in SV1, she would be trying to predict Sally’s choices as the ‘manager’ and that in NSV2, she would be trying to make choices as if she were using the predictions of a computer (cash register). For SV1, Anne was told that the CCTV footage that Sally was receiving was actually uninformative, and for NSV2, Anne was told that the partner shop down the road was closed and so by default the cash register was now receiving misleading information from another partner shop in a completely different place (different island or different city). Finally, Anne was taken to the MEG scanner where she played SV1 followed by NSV2. For various logistical reasons, Sally and Anne did not play their games as the ‘manager’ and ‘shop assistant’ simultaneously, which we explained to Anne. Sally’s responses as the ‘manager’ were saved to a network drive, and they were used to calculate Anne’s score on ‘other’ probe trials in SV1. The same pregenerated trial sequence was given to Sally and Anne except that ‘privileged’ trials were excluded from Sally’s simpleSV1 game—which consisted of only 272 sampling trials—while Anne’s SV1 game consisted of the full 408 sampling trials.

Anne had to play simpleSV1 the previous day for 2 reasons. Firstly, it provided ‘manager’ responses for a previous participant who was playing as the ‘shop assistant’ in the scanner (just like Sally provided ‘manager’ responses while Anne was in the scanner). Secondly, it was to make it more intuitive for Anne to put herself in Sally’s shoes and to understand exactly what information was and wasn’t available to Sally. The reason why Anne had to play simpleNSV2 was simply so that the images and cover stories for the 2 games that Anne played at T2 (SV1 and NSV2) were equally familiar.

### Task implementation

All tasks were implemented in MATLAB (MathWorks, Natick, MA) using Cogent (Wellcome Trust Centre for Neuroimaging, University College London, London, England). All images used were edited in Inkscape and processed in MATLAB to ensure equal luminance (average luminance per pixel) with each other and with the plain grey background.

### Computational models of learning

Our primary hypothesis was that subjects would use PEs to update their beliefs and that they would also simulate the other person’s PEs to solve the belief inference problem. In order to test this hypothesis, we developed a series of computational models to try and explain subjects’ choice behaviours. All the models described here were fitted to choice behaviour from the SV and NSV using identical procedures. We tested 3 different groups of models.

The models in group A assumed that subjects did not use PEs to update their beliefs on each trial but rather used an ‘averaging’ technique. Model 1 predicted a response on each probe trial by taking the average of information sampled since the last probe trial. This model predicted choices on ‘self’ probe trials by averaging information from ‘privileged’ and ‘shared’ trials and predicted choices on ‘other’ probe trials by averaging information from ‘shared’ and ‘decoy’ trials. Model 2 was the same as model 1, but instead of taking the average over the sampling trials since the last probe trial, this model took the average over the last 10 sampling trials.

The models in group B assumed that subjects used PEs to form trial-wise belief updates. All of these models used 2 different PE signals—a PE^s^ (Eq 1) and PE^o^ (Eq 2)—and assumed 2 update equations on each sampling trial, 1 for updating the beliefs of the ‘self’ agent (Eq 3) and 1 for updating the beliefs of the ‘other’ agent (Eq 4). All of the group B models assumed that a nonzero PE^s^ was generated on ‘privileged’ and ‘shared’ trials and that a nonzero PE^o^ was generated on ‘shared’ and ‘decoy’ trials. However, PE^s^ was equal to 0 on ‘decoy’ trials (Eq 1), and PE^o^ was equal to 0 on ‘privileged’ trials (Eq 2).

$${{PE}^{s}}_{\left(t\right)}=\left\{ \begin{array}{cc} 0 & \left(Decoy\right)\\ {Outcome}_{\left(t\right)}-B_{\left(t-1\right)} & \left(Otherwise\right) \end{array}\right.$$ (1)

$${{PE}^{o}}_{\left(t\right)}=\left\{ \begin{array}{cc} 0 & \left(Privileged\right)\\ {Outcome}_{\left(t\right)}-{B_{fb}}_{\left(t-1\right)} & \left(Otherwise\right) \end{array}\right.$$ (2)

where B_(t)_ is the subject’s belief about P on trial t and B_fb(t)_ is subject’s belief about P_fb_ on trial t. All group B models assumed that these estimates were both initialised at 0.5. Outcome was coded as 1 or 0. The simplest models used the following pair of update equations:

$$B_{\left(t\right)}=B_{\left(t-1\right)}+\alpha .{{PE}^{s}}_{\left(t\right)}$$ (3)

$${B_{fb}}_{\left(t\right)}={B_{fb}}_{\left(t-1\right)}+\alpha .{{PE}^{o}}_{\left(t\right)}$$ (4)

where α is the learning rate. This is a free parameter that was fitted to each subject and remained constant throughout the task. It should be noted that when PE^s^ and PE^o^ are zero, B and B_fb_ remain stationary. To account for any information lost since the last update, some models included an additional memory-decay parameter δ, which governs a decay of B and/or B_fb_ back to the initial value of 0.5:

$$B_{\left(t\right)}=B_{\left(t-1\right)}+\alpha .{{PE}^{s}}_{\left(t\right)}+\delta (0.5-B_{\left(t-1\right)})$$ (5)

$${B_{fb}}_{\left(t\right)}={B_{fb}}_{\left(t-1\right)}+\alpha .{{PE}^{o}}_{\left(t\right)}+\delta (0.5-{B_{fb}}_{\left(t-1\right)})$$ (6)

Finally, some models allowed for the possibility that the PE^s^ might be used to erroneously update B_fb_ or that PE^o^ might be used to erroneously update B. Some models assumed a degree of this PE ‘leakage’ with the parameter λ:

$$B_{\left(t\right)}=B_{\left(t-1\right)}+\alpha .{{PE}^{s}}_{\left(t\right)}+\lambda .{{PE}^{o}}_{\left(t\right)}$$ (7)

$${B_{fb}}_{\left(t\right)}={B_{fb}}_{\left(t-1\right)}+\alpha .{{PE}^{o}}_{\left(t\right)}+\lambda .{{PE}^{s}}_{\left(t\right)}$$ (8)

These 3 free parameters α, δ and λ could take any value between 0 and 1. Different models that incorporate various combinations of these parameters can be generated. α can be shared between the 2 update equations, or alternatively, each equation can have its own α with different values. The same can be said for δ and λ, which can also be excluded from either equation entirely. For example, some models included a λ parameter for one update equation but not the other, to allow for a unidirectional PE leak.

The final group of models, group C, had the same update equations as in Eq 5 and Eq 6 (incorporating a shared α and a shared δ), but these models tested the possibility that subjects did not selectively update their beliefs depending on the cues. Model 20 assumed that a nonzero PE^s^ was generated on every sampling trial despite the fact that the information on ‘decoy’ trials should not have been relevant for updating ‘self’. Model 21 assumed that a nonzero PE^o^ was generated on every sampling trial despite the fact that the information on ‘privileged’ trials should not have been relevant for updating ‘other’.

### Data acquisition and preprocessing

MEG was recorded continuously at 600 samples per second using a whole-head 275-channel axial gradiometer system (CTF Omega, VSM MedTech, Coquitlam, Canada) while participants sat upright inside the scanner. Two gradiometers (ML042 and MRC12) were out of service throughout data collection; we preprocessed and analysed data from the remaining 273 channels. We recorded 4 runs of data for each subject (2 runs for the SV and 2 runs for the NSV). Participants made responses on 4 buttons with a button box using the fingers they found the most comfortable. The buttons had the following functions: move arrow left, move arrow right, move arrow faster, and enter choice.

All MEG preprocessing was carried out using the FieldTrip data analysis toolbox [81] on each run of data independently. First, we epoched the data around the relevant triggers and scanned the data for any jump artifacts. In the whole experiment, 1 jump artifact was found, and the relevant trial was excluded from the analysis. Then we downsampled the data from 600 Hz to 100 Hz and filtered the data using a bandpass of 0.5 to 150 Hz and a stopband of 48 to 52 Hz to remove line noise. We then ran an independent components analysis using the built-in ft_componentanalysis function in FieldTrip and manually inspected the components for obvious eye artifacts and cardiac ECG artifacts. The relevant components were removed, and the data were reconstructed. All analyses were conducted on the epoched, filtered, resampled and cleaned data in units of femtotesla.

### Model-free behavioural data analysis

For each subject, on each probe trial, we computed the absolute difference between the subject’s report and the true underlying probability from random walk P or random walk P_fb_. We then computed this difference again for a simulated agent that positioned the arrow at random locations along the response scale. We then subtracted the real difference from the simulated difference on each probe trial and took the mean across probe trials. The resulting value describes how much better than a random subject the real subject performed. We took the mean across subjects to assess group-level performance.

### Model-based behavioural data analysis

All 21 models were fitted to the choice behaviour (on probe trials) of each subject with MATLAB’s nonlinear optimisation function fmincon. We used this function to find the optimal model parameters for each subject as defined by the minimum negative log-likelihood of the subject’s choices conditioned on a set of estimated parameter values. To start the optimisation, every parameter was initialised with a value randomly drawn from a uniform distribution bound by the relevant upper and lower bounds for that parameter. The optimisation procedure was iterated at least 20 times for each model fit, with different initial parameter values each time, to avoid local minima. We selected the iteration with the best-fitting optimised parameters and discarded the rest.

In order to obtain likelihood values, we derived an action likelihood function (ALF) for each trial, which specified the likelihood of any choice that could have been made by the subject on that trial. The subject’s choice could take any value along a continuous scale. Because subjects were technically reporting a probability, this scale was bound between 0 and 1, and we used a Beta distribution to approximate this ALF. Beta distributions are conventionally parameterised by 2 shape parameters, α and β (Eq 9). It should be noted that this is not the same α as the learning rate used in our learning models.

$$f(x;\;\alpha ,\beta )=\frac{x^{\left(\alpha -1\right)}{\left(1-x\right)}^{\left(\beta -1\right)}}{Beta(\alpha ,\beta )}$$ (9)

where *x* is the observed data and *f*(*x; α*, *β*) is the likelihood of that data given the shape parameters of the Beta distribution. The Beta function is a normalisation constant to ensure that the total probability integrates to 1. We wanted to parameterise the ALF with more meaningful parameters: (1) the most likely choice on that trial and (2) the variability or ‘temperature’ of the subject’s actions. We assumed that the most likely choice on a ‘self’ probe trial was the current estimate B_(t)_ and the most likely choice on an ‘other’ probe trial was the current estimate B_fb(t)_, and the mode was assigned to one of these variables (Eq 10). The variance of the Beta distribution was assigned to the value of a free parameter called τ (Eq 12). This parameter was fitted to each subject separately and remained constant throughout the task. It was bound between 0.0001 and 0.08. τ is a temperature parameter that captures how noisy each subject was in his or her mapping from belief to action. A model could have a shared τ parameter for ‘self’ and ‘other’ probe trials or have 2 separate parameters that can vary independently. In order to draw the ALF on each trial, we derived the Beta distribution shape parameters, α and β, which can both be expressed in terms of mode (Eq 11) and variance (Eq 13). These 2 equations could then be solved simultaneously to obtain the shape parameters and then the Beta distribution itself (Eq 9).

$$Mode≔\left\{ \begin{array}{c} B_{\left(t\right)}(Self_{Probe})\\ {B_{fb}}_{\left(t\right)}(Other_{Probe}) \end{array}\right.$$ (10)

$$\frac{\alpha -1}{\alpha +\beta -2}=Mode$$ (11)

$$\sigma^{2}≔\tau$$ (12)

$$\frac{\alpha \beta }{{\left(\alpha +\beta \right)}^{2}(\alpha +\beta +1)}=\sigma^{2}$$ (13)

The real choice that was made by the subject could then be read off the ALF, and the corresponding likelihood would contribute to the joint likelihood of all choices made conditioned on the current model parameter estimates. We computed the BIC for each model and each subject separately (Eq 14) and compared the mean BIC value across subjects for each model to assess relative model evidence. It should be noted that the ALF is a probability density function, and so the likelihoods were often larger than 1. Therefore, the log-likelihoods were often positive and their corresponding BIC values negative.

$$BIC=\ln\left(N\right).k-2ln(\hat{L})$$ (14)

where N is the number of data points that the model was fitted to (in this case, number of trials), k is the number of free parameters in the model, and $\hat{L}$ is the maximised value of the likelihood function of the model.

Using the BIC as an approximation for log model evidence, we also compared the winning model and the second-best model using a hierarchical Bayesian model to estimate the posterior probability that any randomly chosen subject in the sample had data generated by one of those models and not the other. This random-effects Bayesian model selection approach enables us to estimate the parameters *α* of a Dirichlet distribution of the probabilities *r* of the models being compared. These probabilities inform a multinomial distribution over the model space. The hierarchical model is inverted using variational Bayesian approximation [32]. Then, from the Dirichlet parameters, one can compute the expected multinomial parameters 〈*r*_k_〉 for each model k as follows:

$$\langle r_{k}\rangle =\alpha_{k}/(\alpha_{1}+\ldots +\alpha_{k})$$ (15)

One can also compute an exceedance probability *φ*_*k*_, i.e., the belief that a particular model k is more likely than any other model tested, given the group data *y*:

$$\varphi_{k}=p(r_{k}>0.5|y;\alpha )$$ (16)

### Regressing PE magnitude against MEG activity

All analyses were performed on data time locked to the onset of the outcome of the sampling trial because this is the information that subjects would require to generate a PE and update their beliefs. In order to regress PE against recorded brain activity, we performed a mass univariate regression analysis in which we took the absolute PE magnitude as our regressor to ensure we were not capturing brain activity that correlated with the visual attributes of, or other associations with, the image that represented the outcome. We had to take into account the fact that PE^s^ and PE^o^ were coded as 0 on some trials. Therefore, we conducted 2 separate regressions. One regression for PE^s^ excluded ‘decoy’ trials, and 1 regression for PE^o^ excluded ‘privileged’ trials. Therefore, in both regressions, the regressor would contain no systematic pattern of zero-valued elements. If we hadn’t excluded the zero-valued trials, the regression would have been confounded by trial type and cue images (PE^s^ was 0 only after a particular cue image on a ‘decoy’ trial, and PE^o^ was 0 only after a particular cue image on a ‘privileged’ trial).

We regressed PE magnitude against the ERF at each sensor and each time point, to produce a spatiotemporal map of unsigned regression weights. At each sensor, we subtracted the median prestimulus value. We then upsampled the data to create a 95 × 95 2D pixel map of these baseline-corrected effect sizes. Including the time dimension, we ended up with a 3D image for each subject. At the group level, we performed a one-sided Wilcoxon signed-rank test at each pixel in these 3D images to ask whether the group median was significantly greater than 0. We used a nonparametric test here because baseline-corrected unsigned regression weights are not normally distributed. Here, we are testing the null hypothesis that the effect size (unsigned regression weight) is no larger than the prestimulus effect size. We identified points with activations above a cluster-forming threshold (*P* < 0.001). We then identified clusters of contiguous suprathreshold points in this 3D image, which could extend through space and time. We made cluster-level inference by repeating this analysis 300 times, using permuted trial sequences, to generate null distributions of cluster extent, from which we derived significance thresholds (*P* = 0.05 FWE-corrected).

### Classification analyses for agent decoding

We used a nonlinear SVM—LIBSVM for MATLAB [82]—with a radial basis function (RBF) kernel, which was trained on pseudotrial data with 273 features, one for each sensor. Crossvalidation was performed with repeated random subsampling. We performed 200 iterations, and on each iteration, 2 pseudotrials from each class (‘self’ and ‘other’) were randomly sampled and constituted a testing set, while the remainder constituted a training set. On each fold of crossvalidation, the training set and testing set were both centred and normalised with respect to the means and SDs of the data in each feature (MEG sensor) in the training set. Accuracy was measured as the percentage of correct predictions, averaged over the 200 folds of crossvalidation. This value indicated how much information was available in the |PE| signals to discriminate between PE^s^ and PE^o^. We performed this analysis at each point in peristimulus time to generate a time course of CAs. This procedure was conducted once for the SV and again for the NSV.

We tested all classifiers on a range of hyperparameter combinations (regularisation constant C and RBF parameter γ). We optimised each classifier by selecting the combination of hyperparameters that produced the highest CA averaged across subjects. For the |PE| pseudotrial analysis, C = 10^3^ and γ = 10^−6^ in the SV, and C = 10^3^ and γ = 10^−9^ in the NSV. For the ‘signed belief’ pseudotrial analysis, C = 10^3^ and γ = 10^−6^ in the SV, and C = 10^3^ and γ = 10^−6^ in the NSV. For the ‘unsigned belief’ pseudotrial analysis, C = 10^−9^ and γ = 10^−9^ in the SV, and C = 10^−6^ and γ = 10^−9^ in the NSV. The time course of CAs was then smoothed with a moving average filter (moving average span of 10 data points).

We computed significance thresholds by running 150 simulations of these exact analyses, each time with data generated from permuting the trial order. For each permutation, we obtained a maximal CA and used these values to generate a null distribution. If the real CA exceeded the 95th percentile of the null distribution, it was deemed significantly above chance. This method corrects for multiple comparisons in the time domain. We also used this method to determine whether the difference in CA between SV and NSV, at each time point, was significant. For this, we generated a null distribution of maximal (SV − NSV) CA differences and another null distribution of maximal (NSV − SV) CA differences.

### Analysis of questionnaire data

We used scores from the BIDR questionnaire to determine whether any subjects were likely to have a large response bias. This questionnaire allocates a point whenever a subject gives an extreme response (6 or 7 on a 7-point Likert scale) to a question indicating that they might be answering in such a way as to preserve their reputation. No subject had a score more than 2.5 SDs greater than the sample mean, so we had no reason to believe that any subject had an unusually large response bias.

For each subscale of our 5 questionnaires of interest, we set up a regression model with gender and age as predictor variables and the subscale score as a dependent variable. We then took the residuals from these regression models as age- and gender-controlled scores for each subscale. We then z-scored each of these 9 age- and gender-controlled subscales and entered them into a PCA. We investigated the principal component that explained the most variance in the data.

When conducting the correlational analysis at each time point between the PC1 score and the neural agent decoding value, we computed a significance threshold with a permutation-based null distribution. For each of the 150 simulations of the classification analysis, we simulated the correlation analysis to generate a time course of −ln(p) values for each of the 3 types of pseudotrial and then concatenated these 3 time courses together. For each permutation, we took the maximal value of this triple-length time course, resulting in a null distribution of maximal −ln(p) values. The 95th percentile of this distribution represented a significance threshold correcting for multiple comparisons across time and across the 3 types of pseudotrial.

## Supporting information

S1 Data Numerical data for reproducing figures and supporting figures. (XLSX)

S1 Fig Subject performance in behavioural task. Performance of all subjects, relative to chance. Accuracy is measured as distance, in pixels, along the self-report scale. Higher numbers indicate higher accuracy relative to a player who positions the arrow randomly. Chance performance is 0, which would indicate that a random player’s deviation from ground truth is no larger than the subject’s deviation from ground truth. Horizontal bars indicate mean and SEM. There were no significant differences in performance between the different types of probe trials or the different games (SV and NSV). See S1 Data for all numerical values. NSV, nonsocial version; SV, social version.
(PNG)

S2 Fig Qualitative differences between group A and group B models. (A) The red line shows the latent beliefs of a simulated subject who takes the average of the last 10 trials (same as model 2 in group A); the blue line shows the latent beliefs of another simulated subject who uses RW updating on each trial, with a learning rate of 0.1. The group A model simulation has beliefs that change with large steps, whilst the group B model simulation has beliefs that change in smaller gradations. (B) Simulated choice behaviour of the 2 simulated agents in panel A using a temperature parameter of 0.001. The group A model simulation is more likely to overshoot and use the extremes of the scale. (C) Choice behaviour of 2 real subjects. The red line shows a subject who displayed the strongest evidence (relative BIC) for group A models. The blue line shows a subject who displayed weak evidence (relative BIC) for group A models. The behavioural pattern mirrors that shown in the simulations in panel B, with subject 1 using the extremes of the scale more often. See S1 Data for all numerical values. BIC, Bayesian Information Criterion; RW, Rescorla-Wagner.
(PNG)

S3 Fig Parameter recovery. To further test the identifiability and construct validity of the winning model and its parameters, we ran a parameter recovery test. We used model 8 to simulate choice data for each subject and then refitted the model to the simulated data. We assessed the degree of parameter recovery by computing the correlation between true parameter estimates and refitted parameter estimates for each of the 4 parameters and then taking the average of these correlation coefficients. Due to the non-normal distribution of the parameter estimates, we computed nonparametric Spearman’s rank correlation coefficients. The figure shows 2 scatter plots displaying parameter estimates fitted to data simulated by model 8 against parameter estimates fitted to empirical data. Each dot represents a parameter estimate for 1 subject. The SV is on the left, and the NSV is on the right. The parameters fit to the simulated data are highly correlated with the parameters fit to the real data, demonstrating successful parameter recovery. Parameter values are z-scored for display purposes. See S1 Data for all numerical values. NSV, nonsocial version; SV, social version.
(PNG)

S4 Fig Posterior Dirichlet probability density functions from Bayesian model selection. To further quantify the difference between the best and second best models, we used a hierarchical Bayesian model to estimate the posterior probability that one model, and not the other, generated a randomly chosen subject’s data. For the SV (left) and NSV (right), the exceedance probability (probability that model 8 is more likely than model 4) was at least 0.99. α_1_ and α_2_ are the Dirichlet parameter estimates that define the probability density function. *r*_*1*_ is the expected likelihood that model 8 (rather than model 4) generated the data for any randomly chosen subject. *r*_*2*_ is the expected likelihood that model 4 (rather than model 8) generated the data for any randomly chosen subject. See S1 Data for all numerical values. EP, exceedance probability; NSV, nonsocial version; SV, social version.
(PNG)

S5 Fig Patterns of PE encoding in 2 exemplar subjects. Statistical maps (regression effect size) plotted over scalp for 2 subjects at same time points as in Fig 3. There is substantial intersubject heterogeneity in the spatiotemporal patterns of the signals. See S1 Data for all numerical values. PE, prediction error.
(PNG)

S6 Fig Decoding the visual attributes of the cues and outcomes. To make sure the MEG data were clean and appropriate for conducting decoding analyses, we tested to see whether we could decode the visual stimuli used for cues and outcomes. For the SV (left) and NSV (right), we trained a linear SVM on the ERFs in response to cues for ‘privileged’ trials and ‘decoy’ trials (green) and another linear SVM on the 2 outcome stimuli (magenta). We only used data from 94 occipital sensors because here we are exploiting visual information. We trained and tested these classifiers at every time point during a trial. The early and late vertical black bars indicate the onset of the cue and the outcome, respectively. For both the SV and NSV, we could decode the cue image immediately after cue onset, and we could decode both the cue and the outcome immediately after outcome onset. The levels of decoding were similar in the SV and NSV, suggesting that visual discrimination of the stimuli was similar in both games. Shaded regions indicate SEM across 38 subjects. See S1 Data for all numerical values. ERF, event-related field; MEG, magnetoencephalography; NSV, non-social version; SV, social version; SVM, support vector machine.
(PNG)

S7 Fig Spatial topography of neural self–other distinction. Group-level statistical maps of z-scored CAs (frontal sensors towards top of the page). To determine which sensors contributed to neural self–other distinction, we repeated the ‘pseudotrial’ analysis 3,000 times, each time using an independent random subsample of 10 MEG sensors. For each sensor, we found all the samples that included that sensor and calculated the average CA of those samples. This produced a spatial map of CAs, which we averaged across subjects (this method is described in more detail in [83]). On average, left posterior frontal sensors were more implicated for decoding PEs, whilst occipital sensors were more implicated in decoding beliefs. See S1 Data for all numerical values. CA, classification accuracy; MEG, magnetoencephalography; PE, prediction error.
(PNG)

## Abbreviations

ALF: action likelihood function
B: belief about the Bernoulli parameter
B_fb_: belief about the other agent’s false belief about the Bernoulli parameter
BDI: Beck Depression Inventory
BIC: Bayesian Information Criterion
BIDR: Balanced Inventory of Desirable Responding
CA: classification accuracy
CAPE: Community Assessment of Psychic Experience
EEG: electroencephalography; EQ, Empathy Quotient
ERF: event-related field; fMRI, functional magnetic resonance imaging
FWE: family-wise error
ICU: Inventory of Callous-Unemotional traits
IRI: Interpersonal Reactivity Index
ITI: intertrial interval
MEG: magnetoencephalography
mPFC: medial prefrontal cortex
NSV: nonsocial version
P: Bernoulli parameter
P_fb_: the other agent’s false belief about the Bernoulli parameter
PC1: first principal component
PCA: principal components analysis
PE: prediction error
PE^s^: self-attributed prediction error
PE^o^: other-attributed prediction error
RBF: radial basis function
RPE: reward prediction error
RW: Rescorla-Wagner; SV, social version
SVM: support vector machine
