# Supplementary figures and images for: Agent-specific learning signals for self–other distinction during mentalising
Source: PLoS Biol. 2018 Apr 24;16(4):e2004752. doi: 10.1371/journal.pbio.2004752 (PMC5915684; doi:10.1371/journal.pbio.2004752)

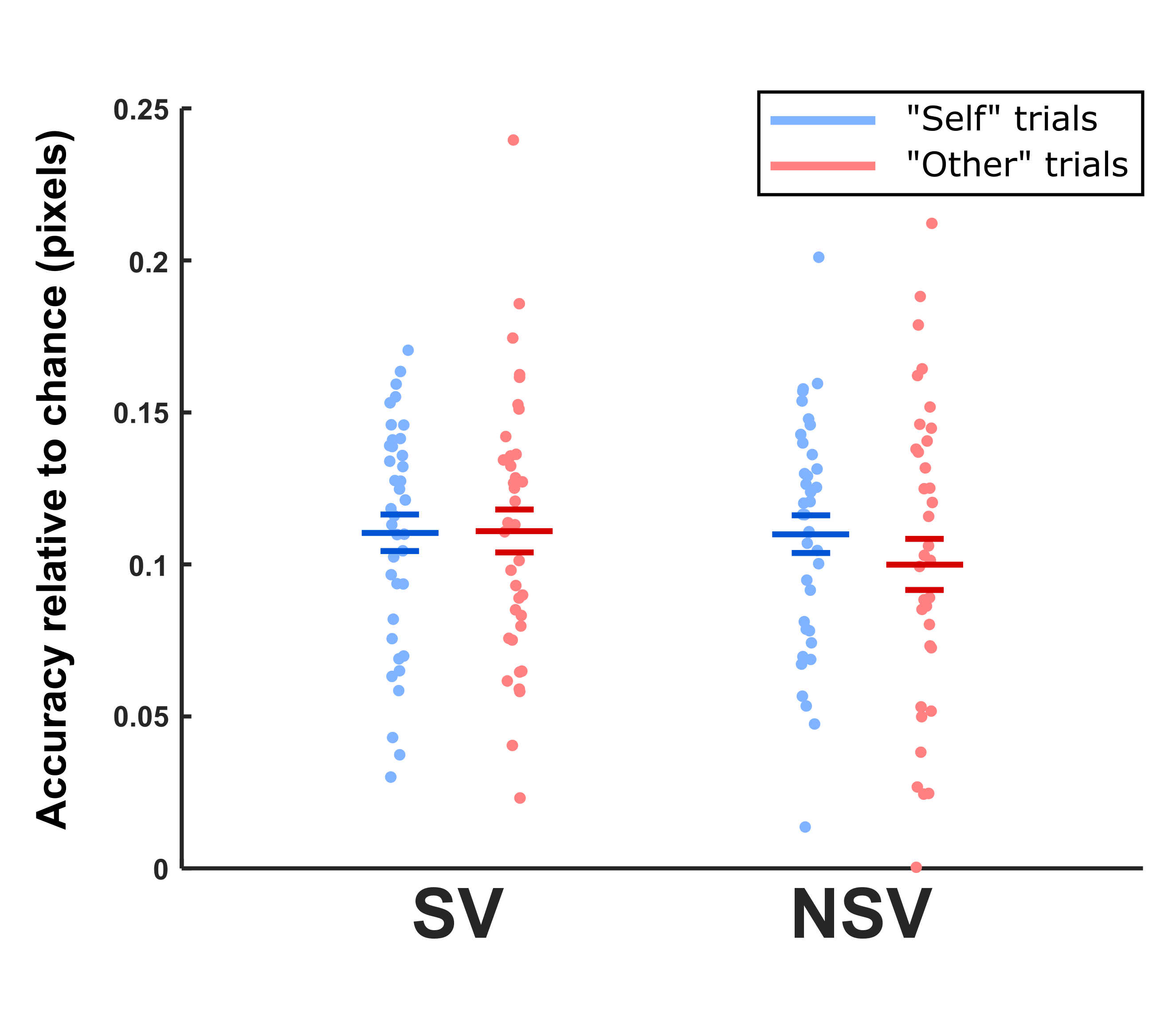

Supplement: S1 Fig — Performance of all subjects, relative to chance. Accuracy is measured as distance, in pixels, along the self-report scale. Higher numbers indicate higher accuracy relative to a player who positions the arrow randomly. Chance performance is 0, which would indicate that a random player’s deviation from ground truth is no larger than the subject’s deviation from ground truth. Horizontal bars indicate mean and SEM. There were no significant differences in performance between the different types of probe trials or the different games (SV and NSV). See S1 Data for all numerical values. NSV, nonsocial version; SV, social version. (PNG) [file pbio.2004752.s002.png]

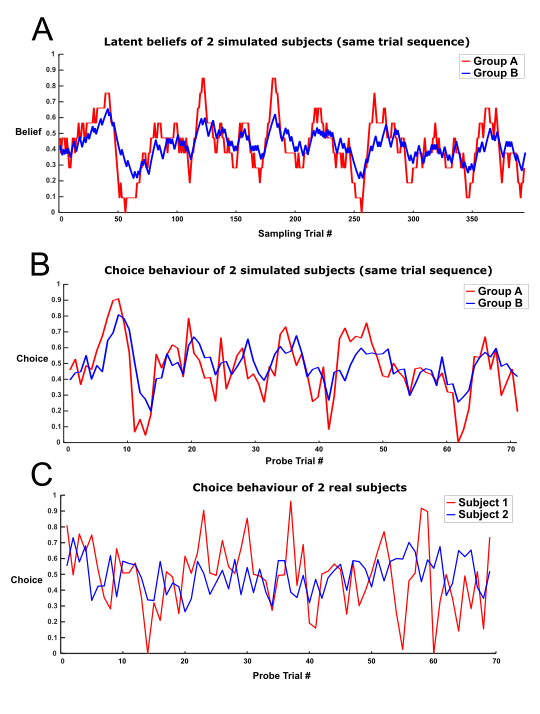

Supplement: S2 Fig — (A) The red line shows the latent beliefs of a simulated subject who takes the average of the last 10 trials (same as model 2 in group A); the blue line shows the latent beliefs of another simulated subject who uses RW updating on each trial, with a learning rate of 0.1. The group A model simulation has beliefs that change with large steps, whilst the group B model simulation has beliefs that change in smaller gradations. (B) Simulated choice behaviour of the 2 simulated agents in panel A using a temperature parameter of 0.001. The group A model simulation is more likely to overshoot and use the extremes of the scale. (C) Choice behaviour of 2 real subjects. The red line shows a subject who displayed the strongest evidence (relative BIC) for group A models. The blue line shows a subject who displayed weak evidence (relative BIC) for group A models. The behavioural pattern mirrors that shown in the simulations in panel B, with subject 1 using the extremes of the scale more often. See S1 Data for all numerical values. BIC, Bayesian Information Criterion; RW, Rescorla-Wagner. (PNG) [file pbio.2004752.s003.png]

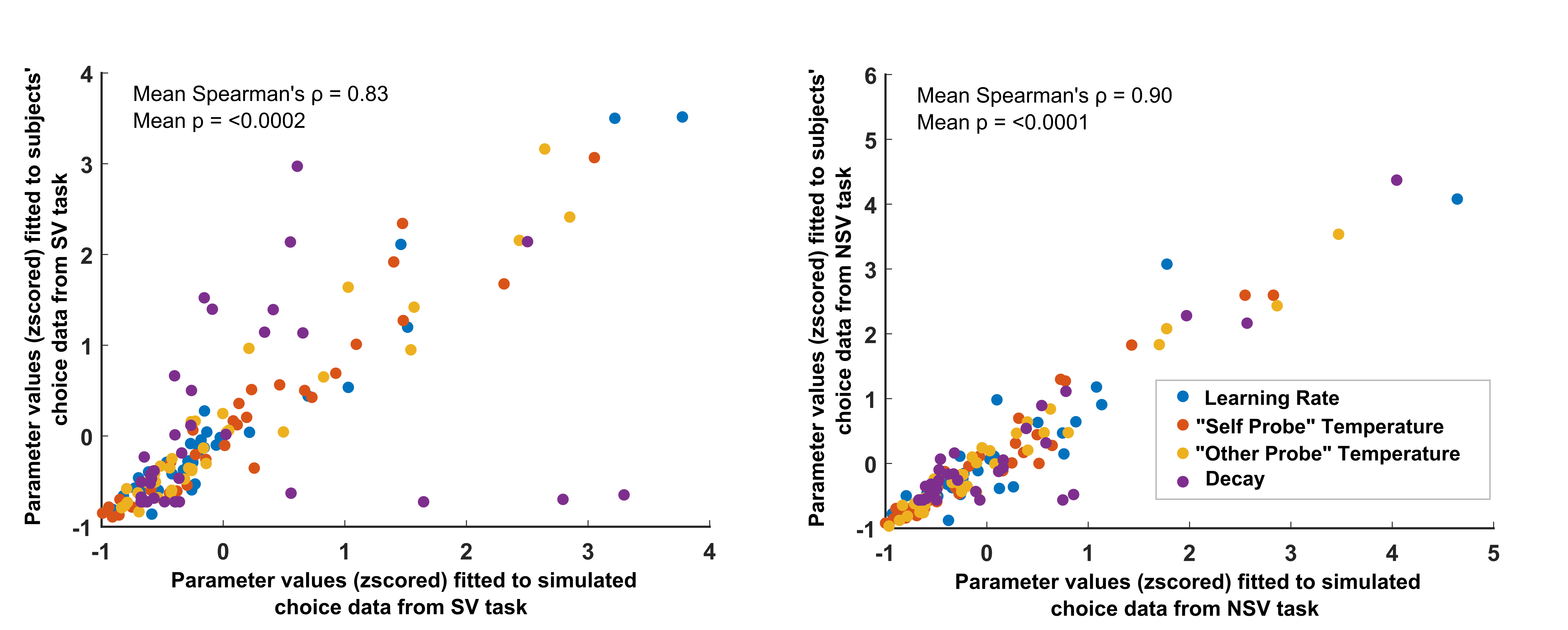

Supplement: S3 Fig — To further test the identifiability and construct validity of the winning model and its parameters, we ran a parameter recovery test. We used model 8 to simulate choice data for each subject and then refitted the model to the simulated data. We assessed the degree of parameter recovery by computing the correlation between true parameter estimates and refitted parameter estimates for each of the 4 parameters and then taking the average of these correlation coefficients. Due to the non-normal distribution of the parameter estimates, we computed nonparametric Spearman’s rank correlation coefficients. The figure shows 2 scatter plots displaying parameter estimates fitted to data simulated by model 8 against parameter estimates fitted to empirical data. Each dot represents a parameter estimate for 1 subject. The SV is on the left, and the NSV is on the right. The parameters fit to the simulated data are highly correlated with the parameters fit to the real data, demonstrating successful parameter recovery. Parameter values are z-scored for display purposes. See S1 Data for all numerical values. NSV, nonsocial version; SV, social version. (PNG) [file pbio.2004752.s004.png]

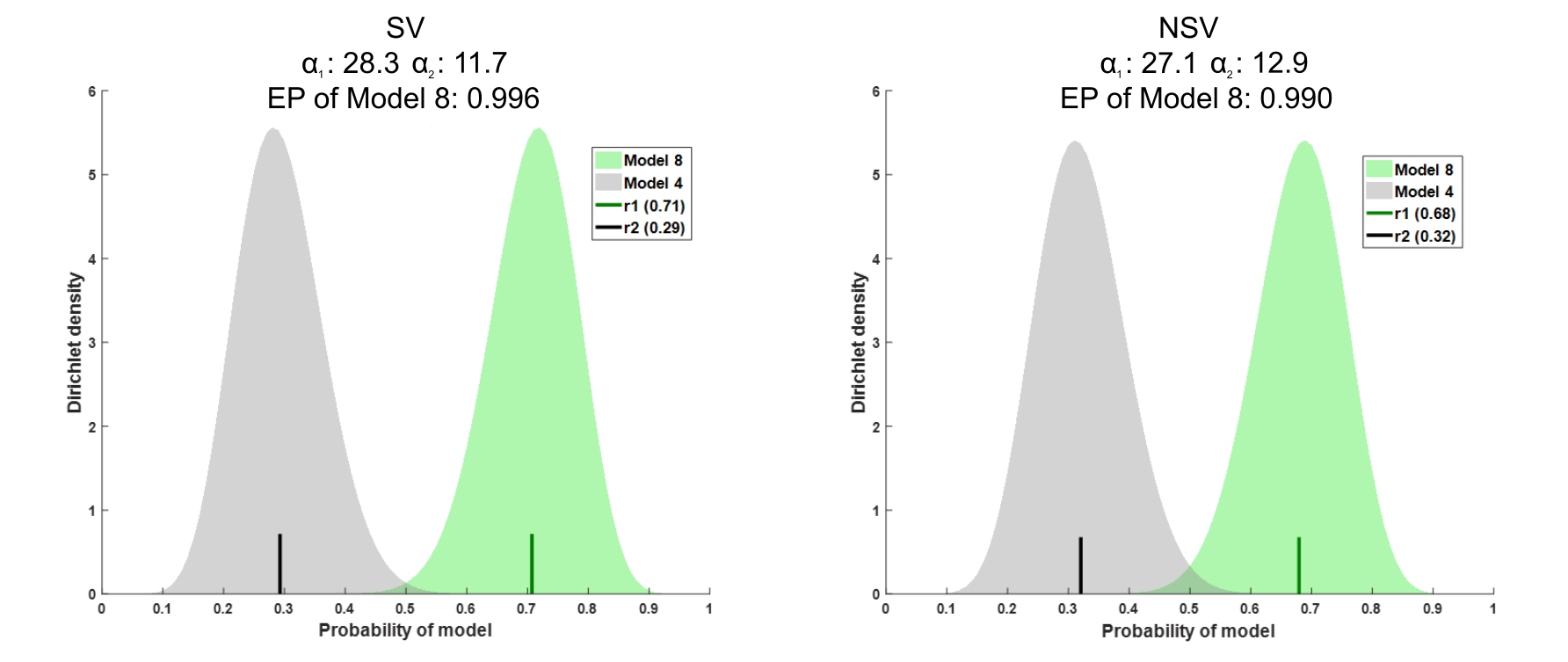

Supplement: S4 Fig — To further quantify the difference between the best and second best models, we used a hierarchical Bayesian model to estimate the posterior probability that one model, and not the other, generated a randomly chosen subject’s data. For the SV (left) and NSV (right), the exceedance probability (probability that model 8 is more likely than model 4) was at least 0.99. α1 and α2 are the Dirichlet parameter estimates that define the probability density function. r1 is the expected likelihood that model 8 (rather than model 4) generated the data for any randomly chosen subject. r2 is the expected likelihood that model 4 (rather than model 8) generated the data for any randomly chosen subject. See S1 Data for all numerical values. EP, exceedance probability; NSV, nonsocial version; SV, social version. (PNG) [file pbio.2004752.s005.png]

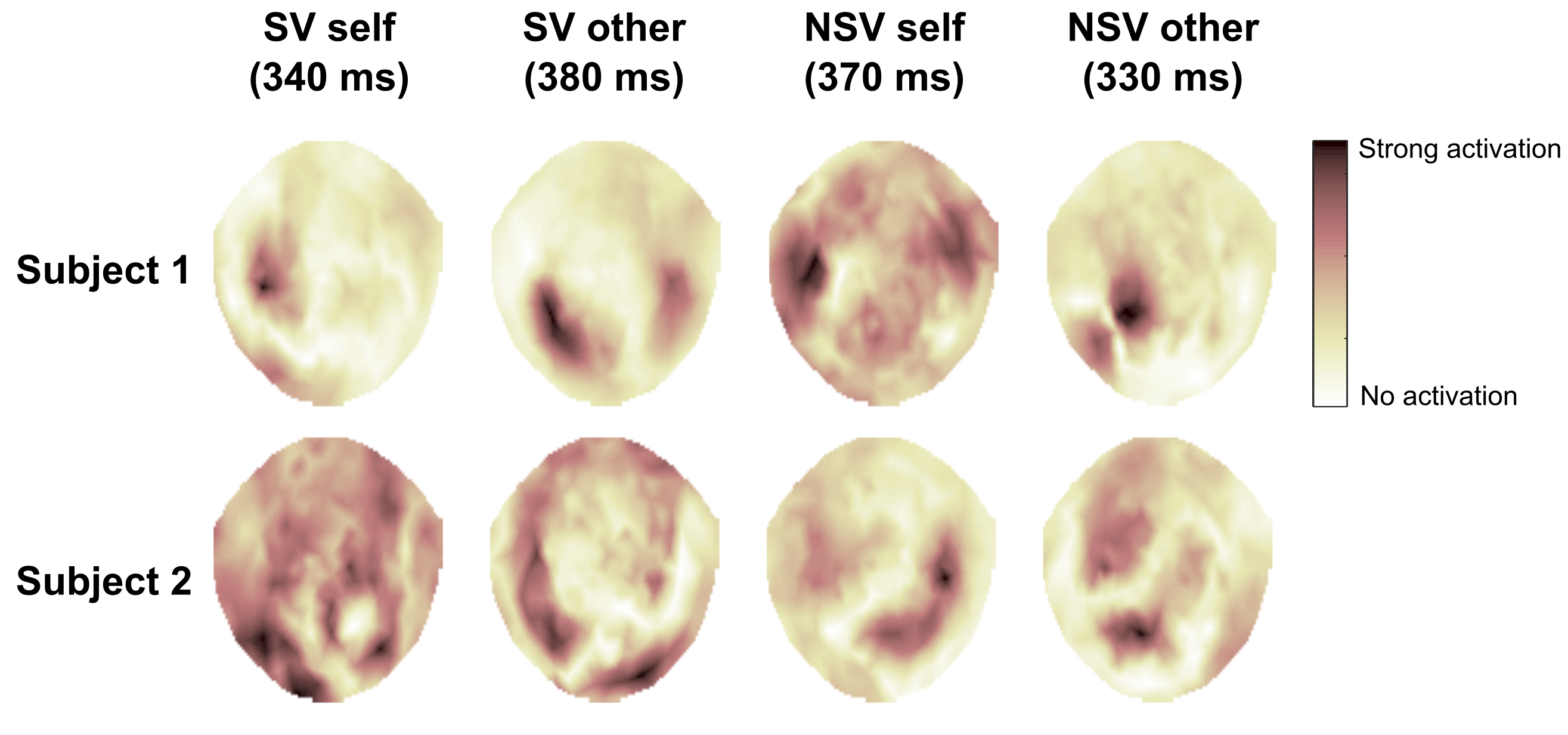

Supplement: S5 Fig — Statistical maps (regression effect size) plotted over scalp for 2 subjects at same time points as in Fig 3. There is substantial intersubject heterogeneity in the spatiotemporal patterns of the signals. See S1 Data for all numerical values. PE, prediction error. (PNG) [file pbio.2004752.s006.png]

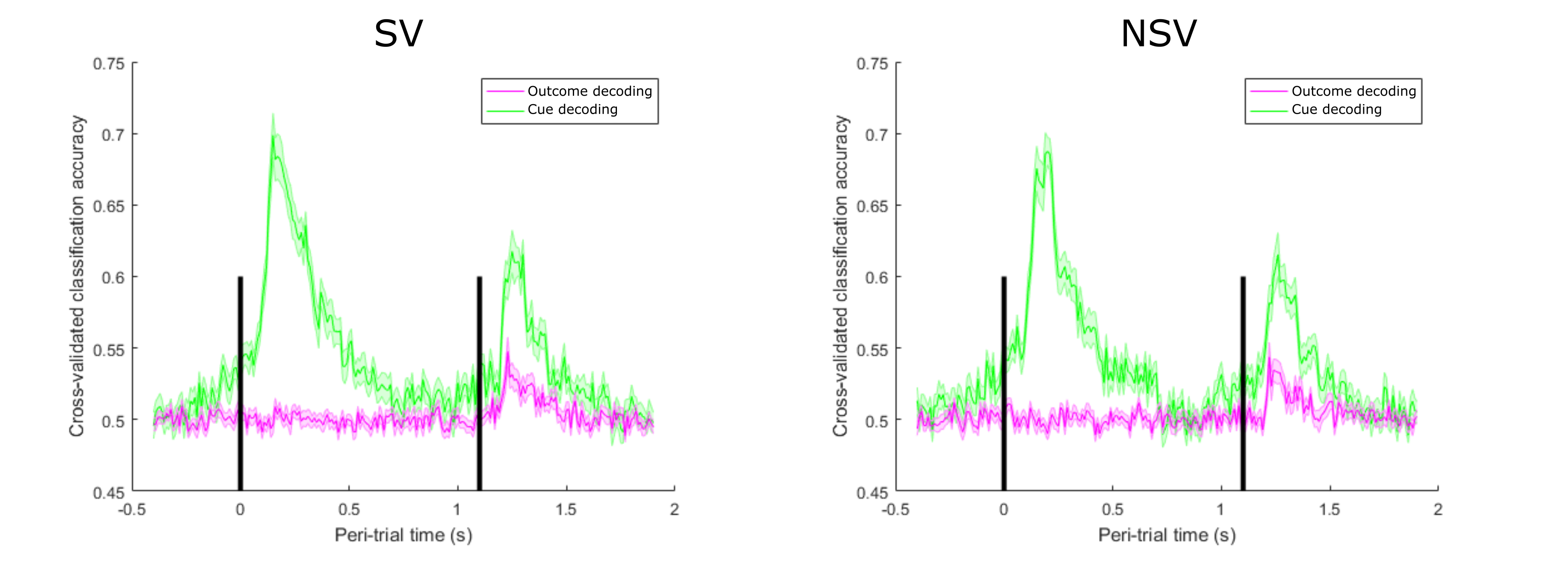

Supplement: S6 Fig — To make sure the MEG data were clean and appropriate for conducting decoding analyses, we tested to see whether we could decode the visual stimuli used for cues and outcomes. For the SV (left) and NSV (right), we trained a linear SVM on the ERFs in response to cues for ‘privileged’ trials and ‘decoy’ trials (green) and another linear SVM on the 2 outcome stimuli (magenta). We only used data from 94 occipital sensors because here we are exploiting visual information. We trained and tested these classifiers at every time point during a trial. The early and late vertical black bars indicate the onset of the cue and the outcome, respectively. For both the SV and NSV, we could decode the cue image immediately after cue onset, and we could decode both the cue and the outcome immediately after outcome onset. The levels of decoding were similar in the SV and NSV, suggesting that visual discrimination of the stimuli was similar in both games. Shaded regions indicate SEM across 38 subjects. See S1 Data for all numerical values. ERF, event-related field; MEG, magnetoencephalography; NSV, non-social version; SV, social version; SVM, support vector machine. (PNG) [file pbio.2004752.s007.png]

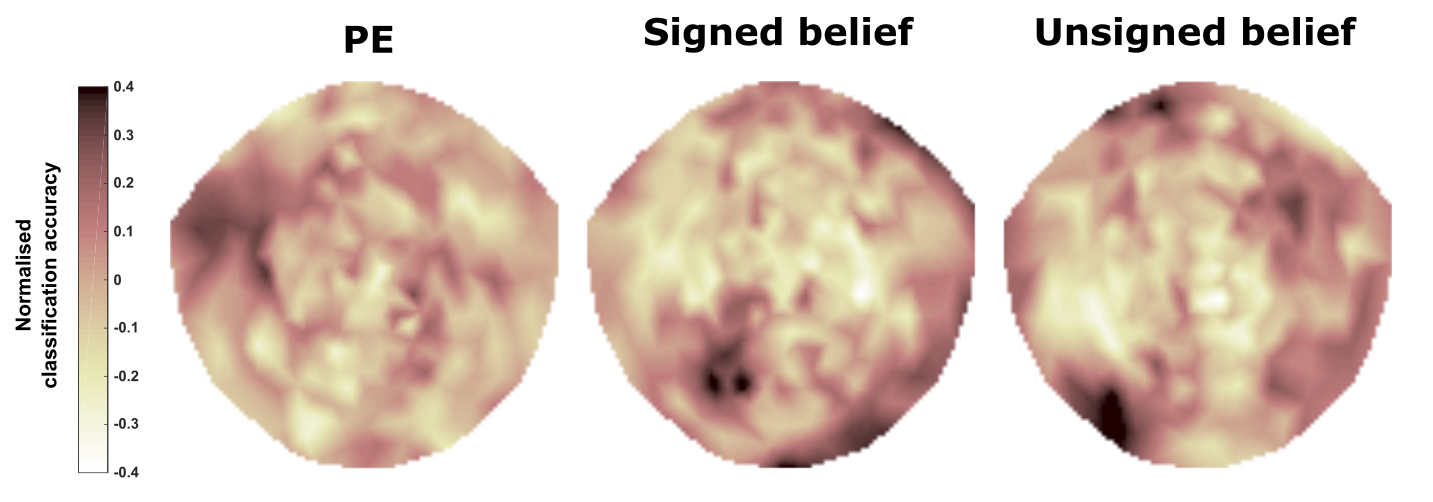

Supplement: S7 Fig — Group-level statistical maps of z-scored CAs (frontal sensors towards top of the page). To determine which sensors contributed to neural self–other distinction, we repeated the ‘pseudotrial’ analysis 3,000 times, each time using an independent random subsample of 10 MEG sensors. For each sensor, we found all the samples that included that sensor and calculated the average CA of those samples. This produced a spatial map of CAs, which we averaged across subjects (this method is described in more detail in [83]). On average, left posterior frontal sensors were more implicated for decoding PEs, whilst occipital sensors were more implicated in decoding beliefs. See S1 Data for all numerical values. CA, classification accuracy; MEG, magnetoencephalography; PE, prediction error. (PNG) [file pbio.2004752.s008.png]
